# *Youngimonas ophiurae* sp. nov., a Quorum-Quenching Marine Bacterium Isolated from a Brittle Star in the South China Sea, and Reclassification of *Lutimaribacter litoralis* as *Youngimonas litoralis* comb. nov.

**DOI:** 10.3390/microorganisms13122661

**Published:** 2025-11-22

**Authors:** Zengzhi Liu, Meng Zhang, Qiliang Lai, Shanshan Xu, Ying Xu

**Affiliations:** 1 Shenzhen Key Laboratory of Marine Bioresource and Eco-Environmental Science, Shenzhen Engineering Laboratory for Marine Algal Biotechnology, College of Life Sciences and Oceanography, Shenzhen University, Shenzhen 518055, China; 2 College of Physics and Optoelectronic Engineering, Shenzhen University, Shenzhen 518060, China; 3 Key Laboratory of Marine Genetic Resources, Third Institute of Oceanography, Ministry of Natural Resources, Xiamen 361005, China

**Keywords:** *Youngimonas*, brittle star, genomic analysis, quorum-quenching

## Abstract

Two novel bacterial strains, designated S70^T^ and S69A, were isolated from a marine brittle star collected in the South China Sea. These strains are Gram-stain-negative, non-motile, aerobic, and rod-shaped. A phylogenomic analysis indicated that strains S70^T^ and S69A formed a distinct branch with *Youngimonas vesicularis* CC-AMW-E^T^ and *Lutimaribacter litoralis* JCM 17792^T^. The DNA G+C content of both strains was 61.5%. The digital DNA–DNA hybridization values with the closest relatives were 21.8, and 21.2%, respectively. Furthermore, the average nucleotide identity (ANIb) values between strain S70^T^ and these two reference strains were 74.9% and 74.6%, respectively, both well below the 95–96% threshold for dividing prokaryotic species. The major fatty acids of strain S70^T^ were summed feature 8 (C_18:1_ *ω*6*c* and/or C_18:1_ *ω*7*c*). Functional genomic analysis revealed that strain S70^T^ possesses potential for hydrocarbon degradation and may play a significant role in sulfur metabolism. Additionally, strain S70^T^ exhibited broad-spectrum AHL-degrading activity and, most notably, significantly inhibited soft rot caused by *Pectobacterium carotovorum* in potato tuber assays. Genomic comparisons further support the reclassification of *Lutimaribacter litoralis* into the genus *Youngimonas*.

## 1. Introduction

In marine ecosystems, particularly in biodiverse regions like the South China Sea, invertebrates harbor diverse microbial communities that contribute to host health, nutrient dynamics, and the production of bioactive metabolites [1,2,3]. Although the South China Sea exhibits high marine biodiversity and unique ecological conditions [4,5], its microbial diversity remains poorly characterized. Most studies of invertebrate-associated microbiota have focused on corals [6,7,8], sponges [9,10,11], sea cucumbers [12,13,14], bryozoans [15,16], and sea anemones [17], with fewer investigations into other invertebrate groups. In our previous study, a total of 197 bacterial strains were isolated from the invertebrate *Onchidium* sp. collected in Dapeng Bay, South China Sea [18]. Among these, five strains were identified and validated as novel species through comprehensive taxonomic analysis [19,20,21,22,23]. Phylogenomic and phenotypic analyses not only confirmed their taxonomic novelty but also elucidated adaptive traits relevant to the intertidal zone. These results highlight the South China Sea as a valuable reservoir of microbial diversity, warranting further systematic exploration of underrepresented marine invertebrates.

Brittle stars (*Ophiuroidea*) inhabit diverse marine environments, from coastal zones to the deep sea, and are notable for their regenerative abilities [24,25]. Recent studies suggest they may also host unique microbial symbionts, though this aspect remains underexplored. Despite their ecological prevalence and intriguing biological traits, the symbiotic bacterial communities associated with brittle stars remain largely uncharacterized. To our knowledge, only one bacterial strain isolated from a brittle star has been taxonomically described to date [26], underscoring a substantial knowledge gap in marine microbial ecology and symbiosis research. Compared to well-studied groups like corals, sponges, and sea cucumbers, brittle stars have received far less attention, underscoring the need for focused research on their microbial communities and host interactions.

During a screening for symbiotic bacteria associated with the brittle star *Ophiactis savignyi* from Qixing bay in the South China Sea, two strains, designated S70^T^ and S69A, were isolated. Preliminary 16S rRNA gene sequence analysis indicated their closest relatives belong to the genus *Thalassovita*, a recently proposed (2023) replacement for the illegitimate genus *Thalassobius* [27], which currently comprises 13 validly published species (https://lpsn.dsmz.de/genus/thalassovita (accessed on 10 October 2025)). However, phylogenomic analysis revealed that strains S70^T^ and S69A form a distinct cluster with members of the genera *Youngimonas* (one valid species) [28] and *Lutimaribacter* (five valid species) [29], both belonging to the family *Roseobacteraceae*. Genomic comparisons also suggested a re-evaluation of the taxonomic placement of *Lutimaribacter litoralis* JCM 17792^T^, as it shared higher average nucleotide identity (ANI) and digital DNA–DNA hybridization (dDDH) values with *Youngimonas* species than with its current genus, reinforcing its potential reassignment to *Youngimonas*. Given the overall taxonomic complexity and close phylogenetic interrelationship among these genera, a comprehensive polyphasic taxonomic study was undertaken.

Acyl-homoserine lactone-based quorum sensing (AHL-QS) is the most well-studied QS mechanism in Proteobacteria [30]. In the recently proposed family *Roseobacteraceae*, approximately 56% of members are predicted to possess AHL-QS systems [31], highlighting the widespread and conserved nature of this trait within the group [32]. Functional genomic analysis of strain S70^T^ identified three lactone biosynthetic gene clusters (BGCs), suggesting not only the potential for AHL production but also the capacity for signal degradation through encoded quorum-quenching (QQ) enzymes, a feature that may contribute to competitive fitness in its symbiotic niche. We therefore propose that this QQ activity likely contributes to the observed inhibition of soft rot disease through disruption of the QS-dependent virulence mechanisms in *Pectobacterium carotovorum* subsp. *carotovorum* (*Pcc*). Given its potent inhibition of this potato pathogen, strain S70^T^ represents a promising candidate for biocontrol.

In this study, we aimed to (i) resolve the taxonomic status of strains S70^T^ and S69A using a polyphasic taxonomic approach that integrated phylogenetic, phylogenomic, phenotypic and chemotaxonomic analyses, (ii) elucidate the ecological role of strain S70^T^ through functional genomic analysis; and (iii) characterize its QQ activity and assess its biotechnological potential. Collectively, our findings highlight the potential of these symbionts as a promising source of novel enzymes, antibacterial agents, and other bioactive molecules, thereby expanding the biotechnological value of echinoderm-associated microbes.

## 2. Materials and Methods

### 2.1. Bacterial Strains and Isolation

Strains S70^T^ and S69A were isolated from the brittle star *Ophiactis savignyi* collected on 6 April 2023, from Qixing Bay (22°33′ N, 114°32′ E), a coastal region of the South China Sea in Shenzhen, China. For strain isolation in July 2023, the brittle star was washed three times with sterile seawater, to remove loosely associated environmental bacteria. The washed specimen was then homogenized using a sterile mortar and pestle. Subsequently, the resulting homogenate was diluted (1:1, *v*/*v*) in a sterile cryoprotective agent containing 70% (*w*/*v*) deionized water, 10% (*w*/*v*) trehalose, and 20% (*w*/*v*) glycerol for preservation. A 20 μL aliquot of the suspension was aseptically spread onto Marine Agar plates (MA, BD Difco™, Sparks, MD, USA) and incubated for 3 days at 28 °C, yielding pure cultures of strains S70^T^ and S69A, which were subsequently maintained on MA medium at 28 °C for routine cultivation. The reference strains *Lutimaribacter litoralis* JCM 17792^T^, *Thalassovita autumnalis* LMG 29904^T^ and *Thalassovita mediterraneus* DSM 16398^T^ were obtained from the Japan Collection of Microorganisms (JCM), LMG Bacteria Collection (LMG), and Deutsche Sammlung von Mikroorganismen und Zellkulturen GmbH (DSMZ), respectively.

### 2.2. 16S rRNA Gene Sequence Analysis

For genomic DNA extraction, cell biomass of strain S70^T^ and S69A was obtained from cultures grown in MB medium at 30 °C for 3 days to late exponential phase. The 16S rRNA gene was amplified by PCR with two universal primers (27F, 5′-AGAGTTTGATCCTGGCTCAG-3′ and 1492R, 5′-GGTTACCTTGTTACGACTT-3′). Then the fragments were ligated into pClone007 Versatile Simple Vector (Tsingke Biotechnology Co., Ltd., Beijing, China). And the genes were sequenced by Beijing Ruibo Xingke Biotechnology Co., Ltd. (Beijing, China). The 16S rRNA gene sequences of strain S70^T^ and S69A comprised 1427 nt., which are almost complete, and the sequences have been deposited in GenBank, the European Nucleotide Archive (ENA) and the DNA Data Bank of Japan (DDBJ; accession number: PV549654 and PV951576).

Sequence homology values between the 16S rRNA genes from strain S70^T^ and S69A and closely related type strains were calculated using the EZBioCloud server (http://www.ezbiocloud.net/ (accessed on 20 April 2024)) [33]. Multiple alignments of the 16S rRNA gene sequence of strain S70^T^ and S69A with those of related strains were performed using the CLUSTAL_X [34]. Phylogenetic trees were constructed by the neighbor-joining (NJ), maximum-likelihood (ML) and maximum-parsimony (MP) algorithms using MEGA12 [35], and were analyzed using bootstrapping [36] based on 1000 re-samplings.

### 2.3. Whole Genome Sequencing and Comparison

Whole-genome sequencing was performed on the Illumina HiSeq PE150 platform. Library construction was conducted by Beijing Novogene Bioinformatics Technology Co., Ltd. (Beijing, China). High-quality paired-end reads were assembled into scaffolds using the SOAP denovo [37,38], followed by gap-closing procedures refine the assembly. The whole genome shotgun projects of S70^T^ and S69A have been deposited at DDBJ/ENA/GenBank under the accession numbers JBNAGJ000000000 (GCF_049803795.1) and JBRAZY000000000 (GCF_052799205.1), respectively.

To elucidate the phylogenomic relationships between strains S70^T^, S69A and their closest relatives, two complementary phylogenomic approaches were employed. A broader-scale phylogenomic analysis was performed with the EasyCGTree pipeline [39] (https://github.com/zdf1987/EasyCGTree4 (accessed on 22 August 2025)), which integrates four marker gene sets (bac120, rhodo268, rp1, and rp2) to construct a supermatrix (SM) for maximum likelihood inference under automatically selected best-fit substitution models using FastTree.

Genomic similarities between strains S70^T^, S69A and their closest relatives were evaluated using pairwise average nucleotide identities (Ortho ANIu and ANIb), calculated via ChunLab’s online ANI calculator and the JSpecies Web Server (JSpeciesWS) [40,41]. Additionally, average amino acid identity (AAI) was calculated using the EzAAI pipeline [42]. Digital DNA-DNA hybridization (dDDH) values were estimated using the Genome-to-Genome Distance Calculator (GGDC) 3.0 (DSMZ) [43], with formula 2 applied for incomplete genomes. The R package pheatmap (version 1.0.12) was used to generate heatmaps with average linkage clustering using Euclidean distance.

### 2.4. Pangenome and Functional Genomics Analysis

Pangenome analysis was conducted using the Integrated Prokaryotic Genome Atlas (IPGA) platform [44] (https://nmdc.cn/ipga/ (accessed on 20 October 2025)) under default parameters. The analysis comprised two main procedures: a pan-genome profiling procedure and a phylogenetic analysis procedure. The draft genome of strain S70T was annotated using the NCBI Prokaryotic Genome Annotation Pipeline (PGAP) [45]. Tandem repeats were identified using Tandem Repeat Finder (TRF version 4.09) [46]. CRISPR arrays were predicted with the web-based tool CRISPRone [47]. Genomic islands were detected with IslandPath-DIMOB (version 1.0.0) [48], and putative prophage regions were predicted using online web server PHASTER [49].

Biosynthetic gene clusters (BCGs) involved in secondary metabolism were predicted by antiSMASH bacterial version 7.1 under default parameters [50]. For metabolic pathway annotation, sulfur metabolism pathways and hydrocarbon degradation pathways were analyzed by querying the KEGG database (release 115.0) using KofamKOALA [51]. The resulting pathways were visualized with KEGG Mapper (https://www.genome.jp/kegg/mapper/reconstruct.html (accessed on 1 June 2024)). The ggplot2 package (version 3.5.2) was employed to create the bubble plot.

Additionally, potential hydrocarbon-degrading genes were identified using the HMMER algorithm in conjunction with the CANT-HYD hidden Markov models [52]. Putative quorum quenching genes were identified by performing BLASTP (version 2.12.0) searches against known reference proteins (*e*-value < 1 × 10^−10^). Sequence similarity networks (SSNs) of these genes were subsequently constructed using the EFI-Enzyme Similarity Tool (EFI-EST) [53], applying an *e*-value cutoffs of 10^−20^ to generate the full networks graphs.

### 2.5. Phenotypic Characterization

Cell morphology was examined using a tungsten filament transmission electron microscope (HT7700, HITACH, Tokyo, Japan) after negative staining with phosphotungstic acid. Growth at various temperatures (4, 10, 12, 15, 20, 24, 28, 30, 37, 40 and 45 °C) was assessed on MA medium over a period 2 weeks to determine the optimal growth temperature and the temperature range supporting growth. To assess the NaCl requirement, bacterial growth was evaluated in modified marine broth medium (MB, BD Difco^TM^, Sparks, MD, USA) containing NaCl concentrations ranging from 0.5% to 10% (*w*/*v*, in 0.5% increments) at 28 °C for 2 weeks. The pH range for growth was determined at 28 °C in MB medium adjusted to pH 2–12 (at intervals of 1 pH unit), using the following buffer systems: Na_2_HPO_4_/citric acid (pH 2.0–6.0), Tris/HCl (pH 7.0–8.0) and Na_2_CO_3_/NaHCO_3_ (pH 9.0–12.0). All pH values were verified after autoclaving. Phenotypic characteristics were assessed using standard methods [54], including catalase and oxidase activities, hydrolysis of starch, DNA, casein, and Tweens (20, 40, 60, and 80), as well as indole and H_2_S production. Enzymatic activities were further analyzed using the API ZYM and API 20NE systems (bioMérieux, Mexico City, Mexico), following the manufacturer’s instructions. Carbon source utilization was assessed using the GEN III MicroPlate (BIOLOG, Cat. No. 1030, Hayward, CA, USA). Antibiotic susceptibility was tested on MA plates using the following antibiotic concentrations: ampicillin (10 μg), amoxicillin (30 μg), carbenicillin (100 μg), ceftriaxone (30 μg), chloramphenicol (30 μg), clarithromycin (15 μg), erythromycin (15 μg), gentamicin (10 μg), imipenem (10 μg), kanamycin (30 μg), lincomycin (15 μg), nalidixic acid (30 μg), neomycin (30 μg), novobiocin (30 μg), penicillin G (10 μg), streptomycin (10 μg), tetracycline (30 μg) and vancomycin (30 μg).

### 2.6. Chemotaxonomic Analysis

The isoprenoid quinone of strain S70^T^ was analyzed by high-performance liquid chromatography (HPLC) following the method of Collins [55]. The analysis was performed on a YMC ODS-A column (4.6 mm× 150 mm) with a mobile phase comprising acetonitrile and isopropanol (3:1, *v*/*v*). Polar lipids of strain S70^T^ were extracted using a chloroform/methanol solvent system and analyzed by one- and two-dimensional thin-layer chromatography (TLC) according to the procedure described by Kates [56]. The analysis was performed on Merck silica gel 60 F254 aluminum-backed plates. For two-dimensional TLC, the plates were first developed with chloroform–methanol–water (65:25:4, *v*/*v*/*v*) and then in the second dimension with chloroform–methanol–acetic acid–water (80:12:15:4, *v*/*v*/*v*/*v*). Total lipid material was visualized using molybdophosphoric acid, while specific lipid classes were identified using selective spray reagents. For cellular fatty acid analysis, cell biomass was collected from strain S70^T^ and three reference strains (*Lutimaribacter litoralis* JCM 17792^T^, *Thalassovita autumnalis* LMG 29904^T^ and *Thalassovita mediterraneus* DSM 16398^T^), and the major fatty acids were analyzed under identical conditions. Fatty acids were saponified, methylated, and extracted following the standard protocol of the MIDI system (Sherlock Microbial Identification System, version 6.0). The analysis was performed by gas chromatography, and fatty acids were identified using the TSBA 6.0 database of the Microbial Identification System [57].

### 2.7. Measurement of AHL Degradative Activity by S70^T^

Strains S70^T^ were pre-cultured in MB medium at 30 °C with shaking until reaching the exponential growth phase (OD_600_ ≈ 0.6). Cells were then harvested by centrifugation (8000 rpm, 10 min), washed three times with 100 μM sterile PIPES buffer (pH 7.0), and resuspended in the same buffer to an OD_600_ of 0.6. For the degradation assay, bacterial suspension was supplemented with various *N*-acyl homoserine lactones (AHLs), including C_6_-HSL, C_8_-HSL, C_10_-HSL, and C_12_-HSL, at a final concentration of 10 μM. The negative control comprised AHLs in PIPES buffer in the absence of bacterial suspension. All reaction mixtures were incubated on a rotatory shaker at 30 °C and 700 rpm for 24 h. Residual AHL was assessed using the reporter strains *Chromobacterium violaceum* CV026 and VIR24 [58,59], and degradation status were determined by measuring the violacein production at OD_550_.

### 2.8. Inhibition of Bacterial-Induced Potato Tuber Decay Assay

The phytopathogenic bacterium *Pectobacterium carotovorum* subsp. *carotovorum* PC1 [60], along with the experimental strain S70^T^, were individually cultured in MB medium with shaking until reaching an OD_600_ ≈ 0.6 (approximately 10^8^ CFU/mL). Bacterial cells were harvested by centrifugation, washed twice, and resuspended in 100 μM sterile PIPES buffer (pH 7.0) to standardize cell density. Subsequently, suspensions of strain S70^T^ and PC1 were mixed at a 1:1 (*v*/*v*) ratio and pre-incubated at 30 °C to facilitate potential bacterial interactions before plant inoculation. Surface-sterilized potato tubers (*Solanum tuberosum* L.) were aseptically cut into uniform discs approximately 1 cm thick. Each disc was inoculated at the center of the exposed surface with 10 μL of the corresponding bacterial suspension according to the following treatment groups: the co-culture of PC1 and S70^T^, PC1 alone (positive control for maceration), and S70^T^ alone (negative control for pathogenicity). All inoculated slices were placed in sterile Petri dishes and incubated at 25 °C in the dark. After incubation, the maceration area on each disc was measured using ImageJ software (version 1.48v). Each treatment was performed in triplicate, and the entire experiment was repeated three times independently.

## 3. Results and Discussion

### 3.1. 16S rRNA Gene Phylogeny

For phylogenetic analyses, the 16S rRNA gene sequences of strains S70^T^ and S69A exhibited similarity values of 96.7%, 96.6%, 96.2% and 96.2% to the type strains *Thalassovita autumnalis* LMG 29904^T^, *Thalassobvita mediterraneus* DSM 16398^T^, *Tritonibacter litoralis* SM1979^T^, and *Aliiroseovarius marinus* A6024^T^, respectively. Notably, the genus *Thalassovita* was recently proposed to replace the illegitimate prokaryotic genus name *Thalassobius* [27]. Consequently, all 14 strains previously classified under *Thalassobius* (https://lpsn.dsmz.de/genus/thalassobius (accessed on 10 October 2025)) were included in the phylogenetic analysis. According to the List of Prokaryotic Names with Standing in Nomenclature (LPSN), 9 of these 14 strains were taxonomically reassigned as follows: three to the genus *Shimia* (*S. byssi* DSM 100673^T^, *S. aestuarii* DSM 15283^T^, and *S. aquaeponti* GJSW-22^T^), two to the genus *Cognatishimia* (*C. active* CETC 5113^T^ and *C. maritima* DSM 28223^T^), and one each to the genera *Ruegeria* (*R. gelatinovorans* corrig. DSM 5887^T^), *Litorimicrobium* (*L. taeanensis* DSM 22007^T^), *Lutimaribacter* (*L. litoralis* JCM 17792^T^), and *Youngimonas* (*Y. vesicularis* CC-AMW-E^T^). Given the high 16S rRNA gene sequence similarity between strain S70^T^ and representatives of the genera *Aliiroseovarius* and *Tritonibacter*—the latter of which includes strains now reclassified into the genus *Epibacterium*—all three genera were included in the comparative analysis.

To resolve the phylogenetic positions of strains S70^T^ and S69A among related genera (*Thalassovita*, *Aliiroseovarius*, *Tritonibacter*, *Epibacterium*, *Lutimaribacter*, *Youngimonas*, *Ruegeria*, *Litorimicrobium*, *Shimia*, and *Cognatishimia*), 16S rRNA gene-based phylogenetic trees were reconstructed using representative type strains. In the neighbor-joining (NJ) tree, strains S70^T^ and S69A formed a robust cluster with the type strains *Thalassovita autumnalis* LMG 29904^T^ and *Thalassovita mediterraneus* DSM 16398^T^ (Figure S1). This clustering pattern was consistently supported by maximum-likelihood (ML) and maximum-parsimony (MP) analyses (Figures S2 and S3). Additionally, these four strains form a large branch with the other three strains (*Thalassovita mangrovi* GS-10^T^, *Thalassovita aquimarina* KMM 8518^T^, and *R. gelatinovorans* corrig. DSM 5887^T^, which is consistent with the ML and MP trees (Figures S1–S3). Collectively, based on 16S rRNA gene sequence alignment and phylogenetic analyses (NJ, ML, and MP trees), strains S70^T^ and S69A showed the closest phylogenetic relationship with the type strains *T. autumnalis* LMG 29904^T^ and *T. mediterraneus* DSM 16398^T^.

### 3.2. Genome-Based Phylogeny

Although 16S rRNA gene phylogeny initially placed strains S70^T^ and S69A within the genus *Thalassovita*, their precise taxonomic classification remains uncertain owing to the high intrafamily diversity of the family *Roseobacteraceae*. This family exhibits extensive genetic and ecological variation, which limits the resolution of 16S rRNA-based phylogeny and has historically resulted in polyphyletic or paraphyletic genera [31]. Therefore, higher-resolution genomic analyses are essential for accurate genus-level identification of strains S70^T^ and S69A.

The genome of strain S70^T^ was assembled into 18 scaffolds with a total length of 3,707,035 bp and an *N_50_* of 444,623 bp, achieving a median read coverage of 364.7x. Similarly, the genome of strain S69A comprised 19 scaffolds totaling 3,684,717 bp, with an *N_50_* of 444,640 bp and a median read coverage of 379.1x. Both genomes have a GC content of 61.5%. Functional annotation of the S70^T^ genome, based on Clusters of Orthologous Groups (COG) categories, is provided in Table S1.

**Figure 1:**
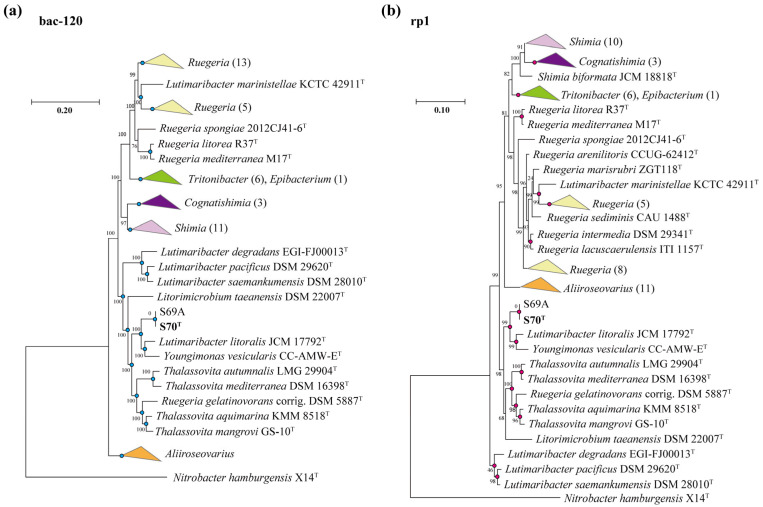
Consensus trees based on the supermatrix (SM) trees of the gene sets bac120 (**a**), and rp1 (**b**), respectively. Blue dots indicate that the branching was supported by two trees (bac120 and rhodo268), pink dots indicate that the branching was supported by two trees (rp1 and rp2).

To complement the 16S rRNA gene phylogeny, we performed a phylogenomic analysis using four core gene marker sets (bac120, rhodo268, rp1, and rp2), including strains S70^T^, S69A, and 65 reference type strains (Figure 1 and Figure S4; genome assembly details are provided in Table S2). The phylogenomic trees revealed a robust clade comprising strains S70^T^, S69A, *Y. vesicularis* CC-AMW-E^T^, and *L. litoralis* JCM 17792^T^ (Figure 1 and Figure S4). This core group further clustered with five additional type strains: *T. mediterraneus* DSM 16398^T^, *T. autumnalis* LMG 29904^T^, *T. mangrovi* GS-10^T^, *T. aquimarina* KMM 8518^T^, and *R. gelatinovorans* corrig. DSM 5887^T^. Notably, this phylogenomic clustering pattern exhibited significant discordance with the 16S rRNA gene-based phylogeny (Figures S1–S3), wherein strains S70^T^ and S69A appeared distantly related to *Y. vesicularis* and *L. litoralis*.

In the broader phylogenetic context, the genus *Lutimaribacter* was polyphyletic. Although *L. degradans*, *L. pacificus*, and *L. saemankumensis* formed a distinct, monophyletic branch, the type strain *L. litoralis* JCM 17792^T^ consistently grouped outside this cluster and instead clustered within the *Youngimonas* clade (Figure 1 and Figure S4). Likewise, *L. marinistellae* KCTC 42911^T^ consistently associated with members of the genus *Ruegeria* (Figure 1 and Figure S4), and *R. gelatinovorans* DSM 5887^T^ clustered within the *Thalassovita* clade (Figure 1 and Figures S1–S4), suggesting that these strains may require taxonomic reclassification.

The consistent and well-supported clustering of S70^T^, S69A, *Y. vesicularis* CC-AMW-E^T^, and *L. litoralis* JCM 17792^T^ across all phylogenomic analyses indicates their close genomic relatedness and supports that their assignment to a single genus.

Crucially, strain S70^T^ demonstrated 100% identity with strain S69A across all genomic metrics (OrthoANIu, ANIb, AAI, and dDDH), confirming their conspecific status. To further elucidate the phylogenetic placement of S70^T^, we performed comparative genomic analyses against 65 type strains. The ANIb-based heatmap revealed clustering with 12 type strains (Figure S5), while the ezAAI analysis displayed a similar clustering pattern, encompassing 11 type strains (Figure S6). General genomic features of strain S70^T^, S69A and these 12 type strains are presented in Table 1. Both S70^T^ and S69A have a DNA G+C content of 61.5%, consistent with that of the other strains, with the exception of *L. pacificus* DSM 29620^T^ (65.5%).

**Figure 2:**
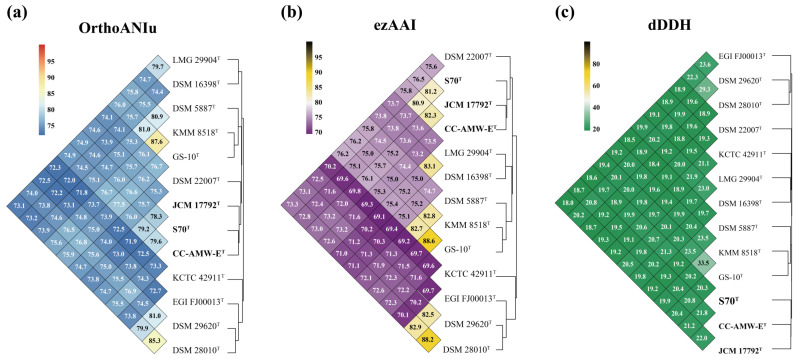
Heatmaps of OrthoANIu (**a**), ezAAI (**b**) and dDDH (**c**) values between strain S70^T^ and 12 close type strains.

Comparison genomic analysis of strain S70^T^ with the 12 type strains using three established indices (OrthoANIu, ezAAI, and dDDH) consistently supported its distinct taxonomic status (Figure 2). OrthoANIu values (72.9–79.6%) and ezAAI values (69.6–81.2%) were all well below respective species delineation thresholds (95–96%) [61], with the highest but still subthreshold affinities to CC-AMW-E^T^ (79.6% OrthoANIu, 80.9% ezAAI) and JCM 17792^T^ (78.3% OrthoANIu, 81.2% ezAAI) (Figure 2a,b). Notably, these two type strains shared higher pairwise similarity (82.3% ezAAI, 22% dDDH) than either showed with S70^T^. The dDDH values (18.6–21.8%) further confirmed this distinction (Figure 2c), being well below the 70% species cutoff [62]. The combined genomic evidence provides definitive support for the classification of strain S70^T^ as representing a novel species. This conclusion is further corroborated by phylogenomic analysis (Figure 1), which consistently places S70^T^ within a distinct phylogenetic lineage. Strain S70^T^ forms a robust clade separate from its closest relatives, strains CC-AMW-E^T^ and JCM 17792^T^, thereby confirming its novel taxonomic status.

### 3.3. Pangenome Analyses

**Figure 3:**
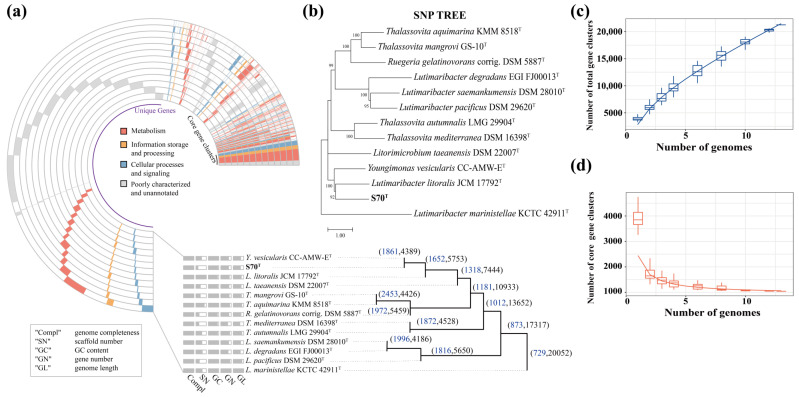
Pangenome analysis of S70^T^ and twelve type strains. (**a**) Pangenome profile showing the functional distribution of core gene clusters and unique genes. The blue numbers indicate the count of genes shared among the different strains; the black numbers indicate the total gene count. (**b**) A single nucleotide polymorphism (SNP)-based tree showing the relationships between S70^T^ and twelve type strains. (**c**) Accumulative curve of the number of total gene clusters and the number of genomes. (**d**) Accumulative curve of the number of core gene clusters and the number of genomes.

Pangenome analysis was conducted to compare functional gene content between strain S70^T^ and twelve type strains (Figure 3). Integrated Prokaryotes Genome and pan-genome Analysis (IPGA) identified 20,052 orthologous genes after genome pooling (Figure 3a). Core gene clusters primarily associated with metabolism, information storage and processing, cellular processes, and signaling, poorly characterized and unannotated (Figure 3a). Among the 20,052 gene clusters, only shared 729 (3.6%) were shared among all thirteen strains (Figure 3a). Strain S70^T^ shared 1861 (42.4%) core gene clusters with strain CC-AMW-E^T^, and these two strains together shared 1652 (28.7%) core gene cluster with strain JCM 17792^T^ (Figure 3a).

A single nucleotide polymorphism (SNP)-based phylogenetic tree further resolved inter-strain relationships, showing S70^T^ clustering most closely with JCM 17792^T^, and subsequently with strain CC-AMW-E^T^ (Figure 3b). Cumulative pangenome curves were generated to assess genomic diversity. The total gene cluster curve continued to rise with each added genome, indicating ongoing incorporation of accessory genes (Figure 3c). In contrast, the core gene cluster curve declined sharply after the first few genomes, reflecting the limited shared gene set, all genome pairs shared fewer than 1600 core gene clusters (Figure 3d). While the SNP phylogeny firmly establishes the close evolutionary relationship among S70ᵀ, JCM 17792ᵀ, and CC-AMW-E^T^, their pangenome architecture reveals significant genomic divergence, suggesting niche-specific adaptation within this clade.

### 3.4. Morphological, Physiological, and Chemotaxonomic Properties

#### 3.4.1. Cellular Morphology

**Figure 4:**
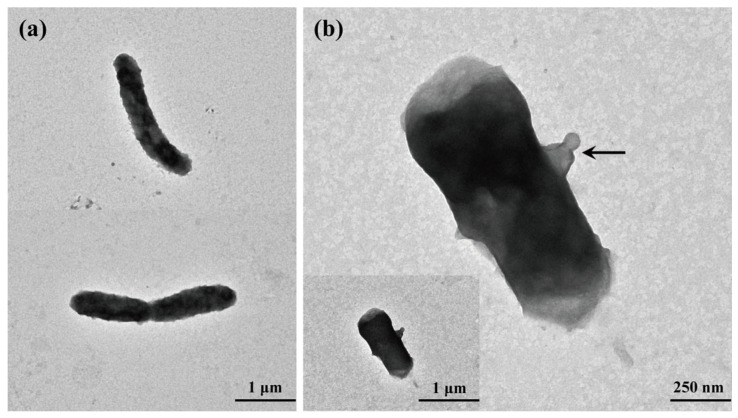
Transmission electron micrograph of negatively stained cells of S70^T^ after incubation in MB for 3 days at 28 °C. (**a**) General cell morphology of S70^T^. (**b**) A detailed view of a vesicle (indicated by the arrow).

Strains S70^T^ and S69A were aerobic, Gram-stain-negative, and non-motile rods (Figure 4a). Cells of S70^T^ measured 0.4–0.7 μm in width and 1.1–5.2 μm in length, with mean dimensions of 0.52 ± 0.22 μm by 2.56 ± 1.57 μm (mean ± SD; *n* = 18). Similarly, cells of S69A ranged from 0.4 to 0.7 μm in width and from 1.6 to 4.1 μm in length, with mean dimensions of 0.57 ± 0.07 μm by 2.32 ± 0.69 μm (mean ± SD; *n* = 18). Notably, a vesicle (approximately 0.2 μm × 0.7 μm) was observed adjacent to a cell of S70^T^ (Figure 4b), a feature also reported in strain CC-AMW-E^T^ [28].

Strain JCM 17792^T^ exhibited pleomorphic rod-shaped cells as previously described [63], whlie strain CC-AMW-E^T^ was also rod-shaped, typically measuring of 2.9–4.3 μm in length and 0.6–0.8 μm in diameter, and frequently occurring as paired rods or filamentous forms up to 8–16 μm long [28]. These observations indicate a close morphological resemblance between the two strains. In contrast, the remaining reference strains displayed distinct morphologies: LMG 29904^T^ displayed a coccobacillary morphology [64], and DSM 16398^T^ showed pleomorphism ranging from coccoid to rod-shaped cells [65]. All strains, including the four reference strains, were non-motile. Morphologically, S70^T^ and S69A are more similar to JCM 17792^T^ and CC-AMW-E^T^.

Based on genomic analyses showing that *L. litoralis* JCM 17792^T^ clusters with strains S70^T^ and CC-AMW-E^T^, we futher analyzed the morphological features of other *Lutimaribacter* species. Most described species are long rods, including *L. saemankumensis* DSM 28010^T^ and *L. degradans* EGI FG00013^T^ [29,66]. In contrast, *L. pacificus* was reported to be short rods, 1.0–1.2 μm long and 0.6–0.7 μm wide [67]. Notably, *L. marinistellae* KCTC 42911^T^ celles are short-rod and motile by means of flagella [68], whereas other *Lutimaribacter* strains are non-motile. Given that motility is observed in some members of the genus *Ruegeria*, suggesting that strain KCTC 42911^T^ require taxonomic reclassification. Collectively, the morphological characteristics of strains S70^T^ and S69A are more similar to strains CC-AMW-E^T^ and JCM 17792^T^.

#### 3.4.2. Physiological and Chemotaxonomic Characteristics

Strains S70^T^ and S69A grew at 12–37 °C, pH 6.0–9.0, and in the presence of 0.5–6.0% (*w*/*v*) NaCl, with optimal growth observed at 28–30 °C, pH 7.0, and 2.0–3.0% (*w*/*v*) NaCl, respectively (Table 2). Both strains were positive for the reduction of nitrate to nitrogen, whereas all four reference type strains were negative for nitrate reduction. In terms of enzyme activity, S70^T^ and S69A were positive for urease, consistent with JCM 17792^T^, but unlike the other three strains, they also hydrolyzed tyrosine, a trait shared with JCM 17792^T^ and LMG 29904^T^, but not with CC-AMW-E^T^ or DSM 16398^T^. Hydrolysis of DNA was observed only in LMG 29904^T^ and DSM 16398^T^, and was absent in the other four strains. Similarly, aesculin hydrolysis was positive only in CC-AMW-E^T^ and LMG 29904^T^, and negative in the remaining strains. Biochemical and enzymatic profiling was performed using the API 20NE and API ZYM systems (bioMérieux) according to the manufacturer’s instructions. The results revealed distinct phenotypic differences among the bacterial strains tested (Table 2). Notably, β-galactosidase activity was detected exclusively to strain LMG 29904^T^. In addition, the ability to oxidize potassium gluconate was unique to strain CC-AMW-E^T^. A shared enzymatic profile, positive for esterase (C4), esterase lipase (C8), and naphthol AS-BI phosphohydrolase, was observed in strains S70^T^, S69A, CC-AMW-E^T^, and JCM 17792^T^.

The results showed that strain S70^T^ and S69A were susceptible to ampicillin, streptomycin, penicillin G, ceftriaxone, clarithromycin, carbenicillin, chloramphenicol, erythromycin, trimethoprim, and polymyxin B. Resistant to kanamycin (w), imipenem, cefalexin, nalidixic acid, neomycin (w), vancomycin, furantoin, gentamicin (w), tetracycline, amoxicillin, lincomycin, fusidic acid, colistin, troleandomycin, rifamycin SV, aztreonam, and minocycline (including BIOLOG GEN III system). BIOLOG GEN III results showed that S70^T^ could utilized dextrin, D-maltose, D-trehalose, gentiobiose, sucrose (weakly), D-turanose, stachyose, D-raffinose, α-D-glucose (weakly), myo-inositol, D-Fructose-6-PO_4_, glycyl-L-proline, L-aspartic acid, L-glutamic acid (weakly), pectin, D-galacturonic acid (weakly), L-galactonic acid (weakly), glucuronamide, *p*-hydroxy-phenylacetic, Tween 40, acetoacetic acid, acetic acid, and formic acid.

In addition, the predominant ubiquinone detected in strain S70^T^ was ubiquinone-10 (Q-10), which was same as other most relative type strains. The major polar lipids of strain S70^T^ were PG, DPG, PE, PME, PC, AL and five unidentified polar lipids (Figure S7). And the major fatty acid (>10%) detected in strain S70^T^ was summed feature 8 (C_18:1_ *ω*6*c* and/or C_18:1_ *ω*7*c*) (75.1%), which is quite similar to the four reference strains (Table S3).

### 3.5. Functional Genomic Analysis

The draft genome sequence of strain S70^T^ was annotated using the NCBI Prokaryotic Genome Annotation Pipeline (PGAP, version 6.10) [45]. Based on the PGAP results, the draft genome of S70^T^ contains 3671 coding sequences, 64 RNAs and 21 pseudogenes. Genome information for the closest related strains can be found in Table S2.

#### 3.5.1. Secondary Metabolite Biosynthetic Gene Clusters (BGCs)

**Figure 5:**
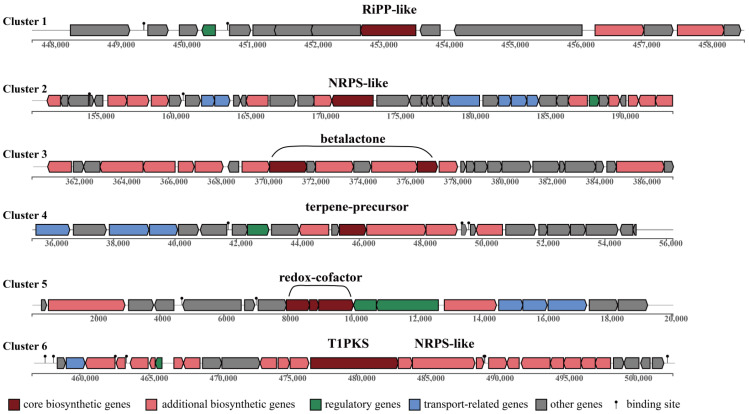
BGC biosynthesis clusters of S70^T^ found in antiSMASH.

The BGCs search results of strain S70^T^ revealed the presence of nine BGCs, which exhibited low homology to known BGCs in MIBiG (Minimum Information about a Biosynthetic Gene cluster) databases, indicating considerable potential for the production of diverse secondary metabolites (Figure 5).

Among the BGCs, the analysis predicted a diverse array of biosynthetic pathways: a ribosomal synthesized and post-translationally modified peptide-like (RiPP-like) cluster (cluster 1); a non-ribosomal peptide synthetase-like (NRPS-like) cluster (cluster 2); a betalactone-related cluster (cluster 3) potentially encoding enzymatically inhibitory molecules; a terpene biosynthesis cluster (cluster 4); and a cluster involved in redox-cofactor metabolism (cluster 5). A particularly noteworthy hybrid cluster (cluster 6) was identified, featuring both type I polyketide synthase (T1PKS) and NRPS-like domains, which significantly expands its potential for generating structurally unique hybrid metabolites (Figure 5).

#### 3.5.2. Hydrocarbon Degradation

Given the reported polycyclic aromatic hydrocarbons (PAH)-degradation activity of *Lutimaribacter degradans* EGI FJ00013^T^ [66], and the close phylogenetic relationship of strain S70^T^ (Figure 1), we characterized its genetic repertoire for hydrocarbon metabolism. Specifically, marker genes associated with hydrocarbon degradation were systematically identified and annotated using the customized HMM (Hidden Markov Model) database of KEGG Orthologs (KOfam) and the Calgary approach to ANnoTating HYDrocarbon degradation genes (CANT-HYD) [52] (Table S4). Genomic analysis of S70^T^ and related strains revealed the presence of both aerobic (AlkB) and anaerobic (AhyA) degradation pathway. This complementary genetic repertoire suggests a broad substrate range and an ability to degrade hydrocarbons under diverse redox conditions.

**Figure 6:**
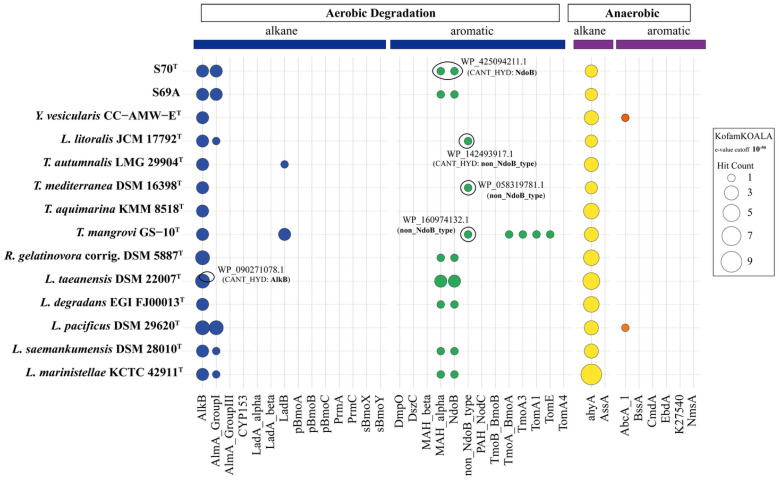
Assessment of hydrocarbon degradation potential in S70^T^ and 12 closely related strains by Hmmer. KofamKOALA HMMs (*e*-value cutoff 10^−50^) and CANT-HYD HMM (scores ≥ noise cutoff) was used to filter results. Each bubble represents the number of KO/CANT-HYD HMM hits (*x*-axis) per genome (*y*-axis), with bubble size corresponding to the number of gene hits. The color-coded bubbles represent genes associated with distinct degradation pathways: blue for aerobic alkane, green for aerobic aromatic, yellow for anaerobic alkane, and orange for anaerobic aromatic degradation. The black circle indicates the hit of CANT-HYD HMM. All HMMs are grouped by substrate type and degradation pathway. Details of the HMMs and underlying data are provided in Tables S4 and S5.

Additionally, genes associated with the aerobic degradation of polycyclic aromatic hydrocarbons (PAHs) were partially identified, including ring-hydroxylating dioxygenases such as MAH_alpha, NdoB, and non_NdoB_type (Figure 6, Table S5). The high CANT-HYD HMM scores of naphthalene dioxygenase components (NdoB and non-NdoB types) in the genomes of strains S70^T^, JCM 17792^T^, DSM 16398^T^, and GS10^T^, strongly suggest their capability to degrade naphthalene and potentially other PAHs. Taken together, this genomic evidence points to a previously unrecognized biodegradation capacity in these strains that merits further experimental investigation.

#### 3.5.3. Sulfite Oxidation and DMSP Degradation Pathways

**Figure 7:**
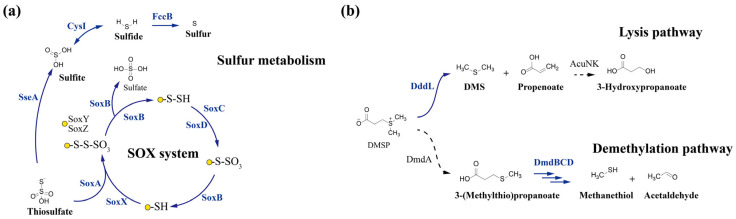
Schematic representation of the sulfur metabolism pathways in strain S70^T^. (**a**) Overview of assimilatory sulfate reduction and organic sulfur metabolism in S70^T^. (**b**) Detailed pathways of dimethylsulfoniopropionate (DMSP) degradation: the cleavage pathway and the demethylation pathway. The solid arrows indicate confirmed enzymatic reactions present in S70^T^; dashed arrows indicate pathways absent or not detected in the strain.

The genomic analysis of S70^T^ reveals a versatile and specialized genetic repertoire for sulfur metabolism (Figure 7, Table S6). The strain harbors a complete sox gene cluster (*soxAXBYZCD*), which encodes the thiosulfate-oxidizing sulfur-oxidizing enzyme (SOX) system, thereby conferring the capacity for inorganic sulfur oxidation and the potential for chemolithoautotrophic growth under energy-limited conditions. In the Sox complex, SoxAX is a heterodimeric c-type cytochrome that mediates electron transfer; *soxB* encodes a hydrolase that catalyzes the hydrolysis of cysteinyl S-thiosulfonate to cysteinyl persulfide and sulfate; *soxCD* encodes the essential sulfur dehydrogenase involved in the reaction mechanism; and *soxYZ* encodes a scaffold protein that binds the substrate (Figure 7a, Table S6). Genes encoding assimilatory sulfite reductase (*cysI*) and thiosulfate sulfurtransferase (*sseA*) were also identified, indicating the strain’s capacity for efficient incorporation of sulfur into biomass (Figure 7a).

Notably, strain S70^T^ exhibits a specialized strategy for organosulfur metabolism, characterized by a genetic predisposition toward dimethylsulfoniopropionate (DMSP) cleavage rather than sulfur assimilation. Although it encodes a DMSP lyase (*dddL*), a trait also reported in related strains CC-AMW-E^T^ and JCM 17792^T^ [69], S70^T^ also possesses an incomplete *dmd* operon, lacking *dmdA* but retaining *dmdBCD*, which is insufficient to support the sulfur-retentive demethylation pathway (Figure 7b). This genomic profile indicates that S70^T^ obligately relies on DddL-mediated cleavage of DMSP to dimethyl sulfide (DMS) and acrylate [70], prioritizing rapid carbon and energy acquisition over sulfur conservation. Consequently, S70^T^ is classified as an obligate producer of DMS—a key climate-active gas—underscoring its potential role in marine sulfur cycling.

The metabolic versatility of strain S70^T^ extends beyond sulfur transformations to encompass pathways for hydrocarbon degradation. The co-occurrence of inorganic sulfur oxidation and organosulfur compound cleavage facilitates adaptation to fluctuating sulfur availability, whereas the concurrent capacity for hydrocarbon degradation likely confers a competitive advantage in the nutrient-rich microenvironment of its brittle star host, *Ophiactis savignyi*. Through these integrated metabolic capabilities, strain S70^T^ may contribute to host energy acquisition and modulate local biogeochemical fluxes of sulfur and carbon in marine ecosystems.

### 3.6. Quorum Quenching Capacity of Strain S70^T^

**Figure 8:**
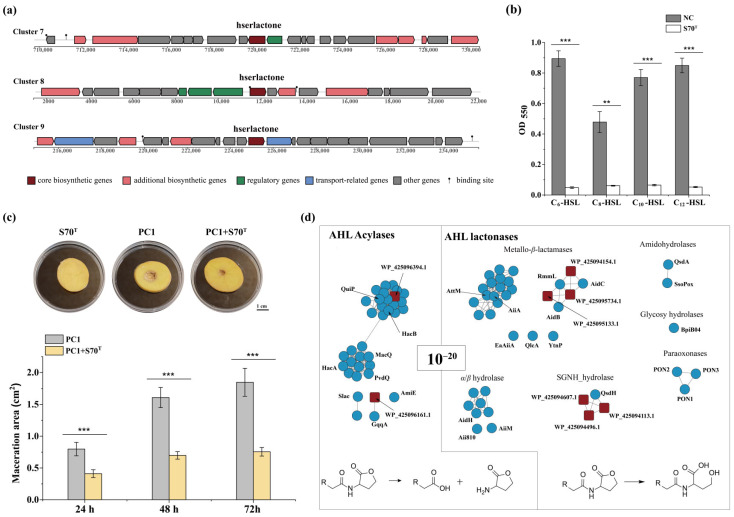
Quorum-quenching (QQ) capacity and anti-phytopathogenic activity of strain S70^T^. (**a**) Identification of putative QS biosynthetic gene cluster (BGCs) in the S70^T^ genome using antiSMASH analysis. (**b**) QQ activity of the S70^T^ culture supernatant against four *N*-acyl homoserine lactones (AHLs), assessed using the biosensor strains *Chromobacterium violaceum* CV026 and VIR24. NC, negative control. (**c**) Biocontrol effect of S70^T^ against soft rot disease caused by *Pcc* on potato tubers. (**d**) Sequence similarity network (SSN) of putative and characterized QQ enzymes (*e*-value < 10^−20^), visualized using Cytoscape (version 3.10.3). Predicted QQ enzymes from the S70^T^ genome are marked with red squares, and known QQ enzyme homologs are indicated with blue circles. Data are represented as the mean ± standard deviation (*n* = 3 biological replicates). Statistical significance was determined using Student’s *t*-test (** *p* < 0.01, *** *p* < 0.001).

Notably, three distinct BGCs (clusters 7, 8, and 9) were annotated as homoserine lactone (AHL) synthases, highlighting a pronounced genetic capacity for quorum-sensing (QS) signal molecule synthesis (Figure 8a). This remarkable abundance of AHL-related BGCs implies that S70^T^ may play a pivotal role in orchestrating microbial communication within its ecological niche.

Building upon the finding that strain S70^T^ possesses an AHL-based quorum sensing (AHL-QS) system, we hypothesized that it may also encode enzymes for AHL degradation, potentially involved in quorum quenching (QQ) to modulate signaling dynamics. This hypothesis was confirmed as strain S70^T^ exhibits broad-spectrum AHL-degrading activity, efficiently utilizing *N*-hexanoyl-L-homoserine lactone (C_6_-HSL), *N*-octanoyl-L-homoserine lactone (C_8_-HSL), *N*-decanoyl-L-homoserine lactone (C_10_-HSL), and *N*-dodecanoyl-L-homoserine lactone (C_12_-HSL) (Figure 8b).

Given that the pathogenic strain *Pectobacterium carotovorum* subsp. *carotovorum* PC1 relies on QS to regulate virulence and maceration enzymes [71], we evaluate the biocontrol potential of strain S70^T^ against potato soft rot. As expected, inoculation with PC1 alone induced severe tissue maceration. In contrast, co-inoculation of PC1 with S70^T^ significantly attenuated the severity of soft rot symptoms (Figure 8c), supporting the conclusion that S70^T^ degrades AHL signals produced by PC1, thereby interfering with its QS-controlled pathogenicity. These results indicate the presence of functional lactonase(s) or acylase(s) responsible for signal inactivation in S70^T^ (Figure 8c).

To identify the underlying QQ enzymes, we analyzed the genome of S70^T^ and identified several candidate proteins: two putative AHL acylases (WP_425096394.1 and WP_425096161.1), three metallo-β-lactamases (WP_425094154.1, WP_425095734.1, and WP_425095133.1), and three RND-type efflux transporter proteins (WP_425094496.1, WP_425094607.1, and WP_425094113.1). To further explore their potential roles, we compared the sequences of these candidate proteins with those of characterized homologs using sequence similarity network (SSN) analysis (Figure 8d, Table S7). In the SSN, WP_425096394.1 clusters with the experimentally validated AHL acylases QuiP [72], HacB [73], and PfmA [74] (all sharing 27% amino acid identity), and belongs to penicillin G acylase family within the N-terminal nucleophile (Ntn) hydrolase superfamily. Protein WP_425096161.1 shares 52% amino acid identity with GqqA, a quorum-quenching enzyme homologous to prephenate dehydratases. The putative AHL lactonases WP_425094154.1, WP_425095734.1, and WP_425095133.1 belong to the metallo-β-lactamase superfamily and cluster in the SSN with the marine bacterium-derived lactonase RmmL [75] (32%, 34%, and 28% identity, respectively) and the terrestrial bacterium-derived lactonase AdiC [76] (23%, 26%, and 25% identity, respectively) (Figure 8d). RmmL has been demonstrated to hydrolyze the lactone ring of a broad range of AHL signal molecules, thereby inactivating quorum-sensing communication [75]. In the SSN, WP_425094496.1, WP_425094607.1, and WP_425094113.1 cluster with QsdH [77] and are annotated as RND-type efflux transporters (Figure 8d). Unlike QsdH, which contains an N-terminal SGNH hydrolase domain responsible for hydrolysis of the AHL ring, these three proteins lack this catalytic domain, suggesting that they are unlikely to exhibit AHL-degrading activity. Given the narrow substrate specificity of AHL acylases for long-chain signals compared to the broad substrate range of lactonases [78], the QQ activity in S70^T^ is likely primarily mediated by lactonases, with acylases playing a secondary role. Nevertheless, their specific QQ activities and biochemical mechanisms require further experimental validation.

The interplay between QS signaling and QQ plays a critical role in the pathogenesis of diseases in marine invertebrates. These invertebrates harbor bacteria capable of producing QQ activities as a defense against pathogen colonization [79]. Strain S70^T^ was isolated from the marine invertebrate brittle star and exhibits QQ activity, suggesting a potential ecological role in maintaining host–microbe homeostasis.

## 4. Conclusions

In this study, two bacterial strains, designated S70ᵀ and S69A, were isolated from a South China Sea brittle star. Phylogenomic analyses consistently placed both strains in a distinct clade along with *Youngimonas vesicularis* CC-AMW-E^T^ and *Lutimaribacter litoralis* JCM 17792^T^. Notably, the JCM 17792^T^ strain did not cluster with other members of genus *Lutimaribacter*, suggesting a misclassification. This phylogenetic positioning was further supported by genomic relatedness indices, including ANI, AAI, and dDDH, which confirmed the close relationship among S70ᵀ, S69A, CC-AMW-E^T^ and JCM 17792^T^. Based on these consistent findings, we propose that both strain S70ᵀ and JCM 17792^T^ should be reclassified under the genus *Youngimonas*. Functional genomic analysis of strain S70ᵀ suggests its potential for polycyclic aromatic hydrocarbon biodegradation and sulfur metabolism, underscoring its diverse metabolic functions in marine ecosystems. Furthermore, the genome harbors numerous putative biosynthetic gene clusters, many of which share limited homology to known clusters. Together, these findings collectively suggest that strain S70ᵀ represents a promising source for discovering novel secondary metabolites. In addition, the strain exhibited inhibitory activity against the plant pathogen *Pectobacterium carotovorum* subsp. *carotovorum*, suggesting the presence of quorum-quenching enzymes. These traits highlight the potential of *Youngimonas* spp. as a promising source of novel bioactive agents, warranting further investigation into their ecological roles and biotechnological potential.

Description of *Youngimonas ophiurae* sp. nov.

*Youngimonas ophiurae* (o.phi.u′rae. N.L. gen. n. *ophiurae* of Ophiura, a class of invertebrates belonging to the Ophiuroidea, the source of isolation of the type strain).

The detailed description is provided in the Supplementary Material (see Description of *Youngimonas ophiurae* sp. nov.). The type strain, S70^T^ (=KCTC 8975^T^ = MCCC 1K09707^T^), was isolated from a brittle star collected in the South China Sea, Shenzhen, China. G+C content is 61.5%. The GenBank accession numbers for the type strain are JBNAGJ01 (genome) and PV549654 (16S rRNA gene).

Description of *Youngimonas litoralis* comb. nov.

Basonym: *Lutimaribacter litoralis* Iwaki et al. 2013 [63]. The description is as given for *L. litoralis* [63]. Genomic, phylogenetic, and phenotypic evidence strongly support the placement of this species in the genus *Youngimonas*. The type strain is KU5D5^T^ (=JCM 17792^T^ = KCTC 23600^T^). G+C content is 59.1%. The GenBank accession numbers for the type strain are FXTO01 (genome) and AB627076 (16S rRNA gene).

## Figures and Tables

**Table 1:** General genomic features of strains S70^T^, S69A and the closest type strains.

Strains		1	2	3	4	5	6	7	8	9	10	11	12	13	14
Genomic size (Mb)		3.7	3.7	3.8	4.1	4.4	3.4	4.8	4.2	4.0	4.0	3.6	4.4	3.8	5.1
GC (%)		61.5	61.5	63.5	59.0	60	58.5	62.5	64	58.5	60.5	63	65.5	63	62
Protein		3671	3648	3694	3957	4133	3218	4157	3965	3775	3822	3448	4292	3741	4639
Gene		3757	3734	3789	4102	4212	3294	4260	4027	3859	3919	3545	4375	3842	4760
rRNA		12	12	5	3	3	3	3	4	5	5	3	3	33	4
tRNA		49	49	47	48	54	46	44	44	44	42	45	43	46	44
Other RNA		3	3	3	3	3	3	3	3	3	3	3	3	3	3
Pseudogene		21	21	40	91	19	24	52	10	31	47	46	34	49	70
Tandem repeats		106	106	94	49	83	62	123	168	80	112	123	126	70	82
Genomic islands		1	1	0	0	0	0	0	1	0	0	0	1	0	0
Prophage		5	4	4	5	4	1	2	1	5	4	2	3	2	3
CRISPR System	Number	0	0	2	0	1	1	4	1	0	2	0	1	0	1
	cas gene	–	–	3/3	–	4	0	2/4/0/3	3	–	3/3	–	9	–	3
	Type	–	–	NA	–	NA	NA	NA	NA	–	NA	–	I	–	NA

Strain: 1, S70^T^; 2, S69A; 3, *Y. vesicularis* CC-AMW-E^T^; 4, *L. litoralis* JCM 17792^T^; 5, *T. autumnalis* LMG 29904^T^; 6, *T. medi-terranea* DSM 16398^T^; 7, *T. aquimarina* KMM 8518^T^; 8, *T. mangrovi* GS-10^T^; 9, *R. gelatinovora* corrig. DSM 5887^T^; 10, *L. taeanensis* DSM 22007^T^; 11, *L. degradans* EGI FJ00013^T^; 12, *L. pacificus* DSM 29620^T^; 13, *L. saemankumensis* DSM 28010^T^; 14, *L. marinistellae* KCTC 42911^T^.

**Table 2:** Differential characteristics between strain S70^T^, S69A and their phylogenetically closest type strains.

Characteristic	1	2	3 ^a^	4	5	6
Colony color on MA	Pale yellow	Pale yellow	Ivory-colored	Pale yellow ^b^	Brown diffusible pigment ^c^	Non-pigmented ^d^
Cell shape	Rod shaped	Rod shaped	Rod shaped	Pleomorphic rods ^b^	Coccobacillary shaped	Coccoid to rod shaped ^d^
Cell size (μm)	0.4–0.6 × 1.1–5.2	0.4–0.7 × 1.6–4.1	0.6–0.8 × 2.9–4.3	0.4–1.2 × 2.0–at least 10.0 ^b^	NR	0.5–0.8 × 0.5–2.0 ^d^
Growth temperature (Optimal)	12–37 °C (28–30 °C)	12–37 °C (28–30 °C)	20–37 °C (30 °C)	10–37 °C (24–30 °C)	15–37 °C (24–28 °C)	12–37 °C (24–28 °C)
NaCl (%) (Optimal)	0.5–6 (2–3)	0.5–6 (2–3)	1–10 (NR)	0.5–6 (2–4)	1–5.5 (2)	1–5.5 (2–4)
pH (Optimal)	6–9 (7–8)	6–9 (7)	6–9 (7)	6–9 (7)	6–9 (7)	6–9 (7)
Nitrate reduction	N_2_	N_2_	−	−	−	−
Hydrolysis of						
Tween 20	w	NG	−	NG	w	NG
Tween 40	+	+	NR	+	w	+
Tween 60	+	−	NR	+	−	+
Tween 80	−	NG	−	−	−	−
DNase	−	−	–	−	+	+
Aesculin	−	−	+	−	+	−
Gelatin	−	−	w	−	−	−
L-tyrosine	+	+	−	+	+	−
Urea	+	w	−	w	−	−
Assimilation tests (API 20NE)						
PNPG	−	−	−	−	+	−
Potassium gluconate	−	−	+	−	−	−
Enzyme activity (API ZYM)						
Alkaline phosphatase	w	+	+	−	−	−
Esterase (C4)	+	+	+	+	−	−
Esterase lipase (C8)	w	+	+	+	−	−
Acid phosphatase	−	−	+	−	−	−
*α*-Chymotrypsin	−	−	+	−	−	−
*N*-acetyl-glucosaminase	−	w	−	−	−	−
Naphthol-AS-BI-phosphohydrolase	+	+	+	+	−	+

^a^ Data from previous study [28]; ^b^ Data from previous study [63]; ^c^ Data from previous study [64]; ^d^ Data from previous study [65]. Strains: 1, S70^T^; 2, S69A; 3, *Y. vesicularis* CC-AMW-E^T^; 4, *L. litoralis* JCM 17792^T^; 5, *T. autumnails* LMG 29904^T^; 6, *T. mediterraneus* DSM 16398^T^; all data were generated in this study unless mentioned. All of these strains are Gram-stain-negative and non-motile; positive for activities of oxidase, catalase and leucine arylamidase. All of these strains are negative for lipase (C14), arginine dihydrolase, valine arylamidase, cystine arylamidase, α-glucosidase and β-galactosidase, hydrolysis of starch and casein, production of indole and H_2_S; do not ferment carbohydrates (glucose, arabinose, maltose, mannitol, mannose, *N*-acetylglucosamine, capric acid, adipic acid, malic acid, citric acid and phenylacetic acid). +, positive; −, negative; w, weakly positive; NR, not reported; NG, no growth.

## Data Availability

The authors declare that all relevant data supporting the findings of this study are available within the article. The whole genome shotgun sequencing projects for S70^T^ and S69A have been deposited in the DDBJ/ENA/GenBank databases under the accession numbers JBNAGJ000000000 and JBRAZY000000000, respectively. The accession numbers for the 16S rRNA gene sequences of strain S70^T^ and S69A are PV549654 and PV951576, respectively.

## Supplementary Materials

The following supporting information can be downloaded at: https://www.mdpi.com/article/10.3390/microorganisms13122661/s1, Figure S1: Neighbor-joining phylogenetic tree of 16S rRNA gene sequences, showing the position of strains S70T and S69A among representatives of the Roseobacteraceae; Figure S2: Maximum-likelihood phylogenetic tree of 16S rRNA gene sequences, showing the position of strains S70T and S69A among representatives of the Roseobacteraceae; Figure S3: Maximum-parsimony phylogenetic tree of 16S rRNA gene sequences, showing the position of strains S70T and S69A among representatives of the Roseobacteraceae; Figure S4: Consensus trees based on the super-matrix (SM) trees of the gene sets rhodo268 and rp2, respectively; Figure S5: The pyANIb heatmap with hierarchical clustering was constructed using genomic data retrieved from the NCBI database; Figure S6: The ezAAI-based heatmap with hierarchical clustering was generated using genomic data obtained from the NCBI database; Figure S7: Polar lipids of strain S70T separated by two-dimensional TLC; Table S1: Number of genes associated with the general COG functional categories for strain S70T; Table S2: Genome assembly numbers of the strains used in this study; Table S3: Cellular fatty acid composition (%) of strain S70T and its phylogenetically closest strains. Table S4: Number of marker genes associated with hydrocarbon degradation; Table S5: Genes associated with hydrocarbon degradation; Table S6: Key genes functioning in sulfite oxidation and DMSP degradation pathways across S70T and related strains; Table S7: Putative quorum quenching proteins in strain S70T and previously characterized QQ enzymes.

## Abbreviations

The following abbreviations are used in this manuscript:

ANI	Average Nucleotide Identity
AAI	Average Amino Acid Identity
dDDH	Digital DNA–DNA hybridization
BGC	Biosynthetic Gene Cluster
LPSN	List of Prokaryotic Names with Standing in Nomenclature
AHL	Acyl-Homoserine Lactone
QS	Quorum Sensing
QQ	Quorum Quenching
NJ	Neighbor-Joining
ML	Maximum-Likelihood
MP	Maximum-Parsimony
CANT-HYD	Calgary approach to ANnoTating HYDrocarbon Degradation Genes
NRPS-like	Nonribosomal Peptide Synthetase-Like
RiPP-like	Ribosomal Synthesized and Post-Translationally Modified Peptide-Like
T1PKS	Type I Polyketide Synthase
DMS	Dimethylsulfide
DMSP	Dimethylsulfoniopropionate
HMM	Hidden Markov Model
PAH	Polycyclic Aromatic Hydrocarbons
SSN	Sequence Similarity Network
