# Supplementary material for: Youngimonas ophiurae sp. nov., a Quorum-Quenching Marine Bacterium Isolated from a Brittle Star in the South China Sea, and Reclassification of Lutimaribacter litoralis as Youngimonas litoralis comb. nov
Source: Microorganisms. 2025 Nov 22;13(12):2661. doi: 10.3390/microorganisms13122661 (PMC12735020; doi:10.3390/microorganisms13122661)
Supplement: Supplementary file 1 [file microorganisms-13-02661-s001.zip › Supplementary materials-Figure S1-S7-Table S1-S3.pdf]

Supplementary materials

Youngimonas ophiurae sp. nov., a quorum-quenching marine bacterium isolated from a brittle star in the South China Sea, and reclassification of *Lutimaribacter litoralis* as *Youngimonas litoralis* comb. nov.

Zengzhi Liu[1, 2], Meng Zhang[1], Qiliang Lai[3], Shanshan Xu[1, *], Ying Xu[1]

[1] Shenzhen Key Laboratory of Marine Bioresource and Eco-Environmental Science, Shenzhen Engineering Laboratory for Marine Algal Biotechnology, College of Life Sciences and Oceanography, Shenzhen University, Shenzhen 518055, PR China

[2] College of Physics and Optoelectronic Engineering, Shenzhen University, Shenzhen 518060, PR China

[3] Key Laboratory of Marine Genetic Resources, Third Institute of Oceanography, Ministry of Natural Resources, Xiamen 361005, PR China

***Correspondence: xushanshan328@163.com**

Supplementary Figures

Supplementary Figure 1 Neighbor-joining phylogenetic tree of 16S rRNA gene sequences, showing the position of strains S70^{T} and S69A among representatives of the *Roseobacteraceae*. Bootstrap values (above 50%) based on 1000 re-samplings are shown at branch nodes. Hollow squares indicate the clades that were conserved in the maximum likelihood and maximum parsimony trees. Hollow circles indicate the clades that were conserved in the maximum likelihood or maximum parsimony trees. *Nitrobacter hamburgensis* X14^{T} (GenBank accession number, L11663) was used as an outgroup. Bar, 0.02 substitutions per nucleotide position.

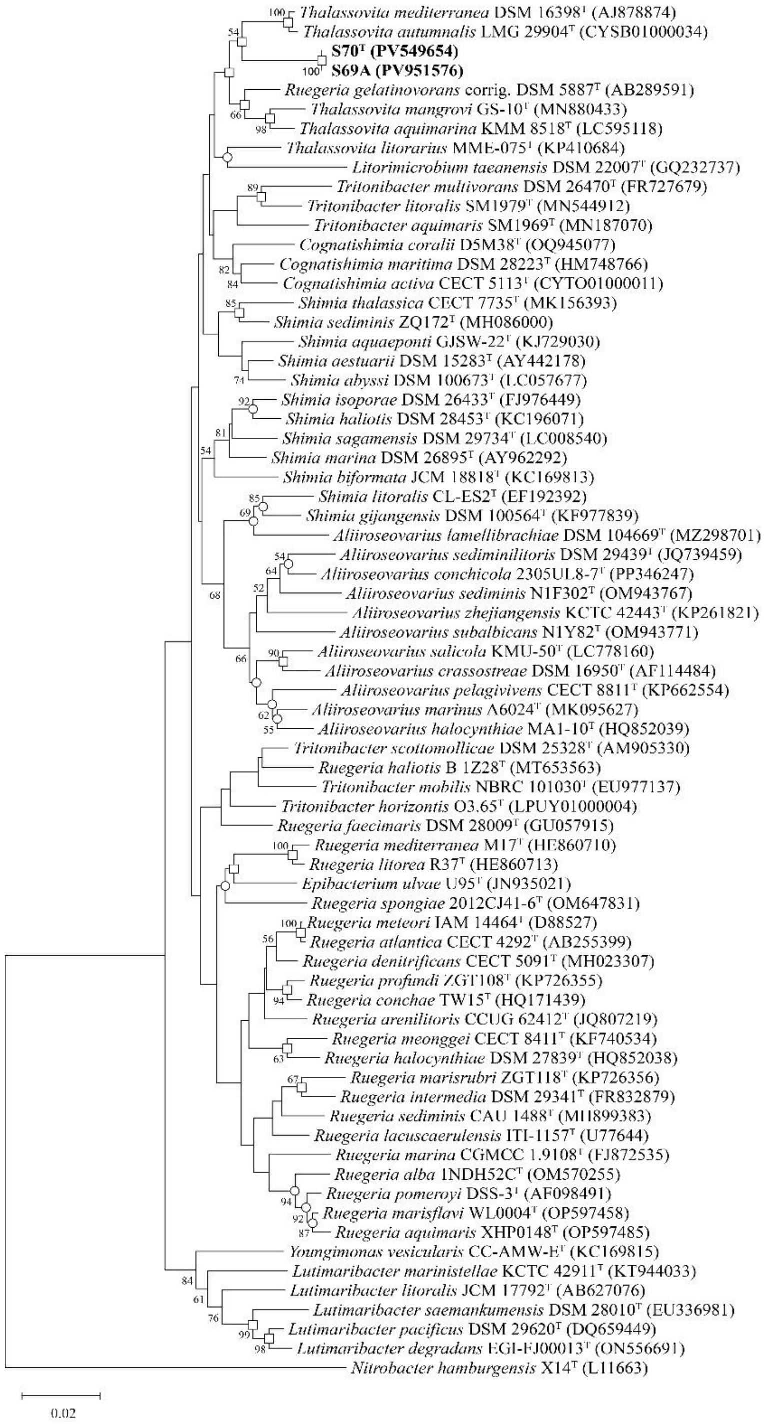

Supplementary Figure 2 Maximum-likelihood phylogenetic tree of 16S rRNA gene sequences, showing the position of strains S70T and S69A among representatives of the *Roseobacteraceae*. Bootstrap values (above 50%) based on 1000 re-samplings are shown at branch nodes. *Nitrobacter hamburgensis* X14^{T} (GenBank accession number, L11663) was used as an outgroup. Bar, 0.05 substitutions per nucleotide position.

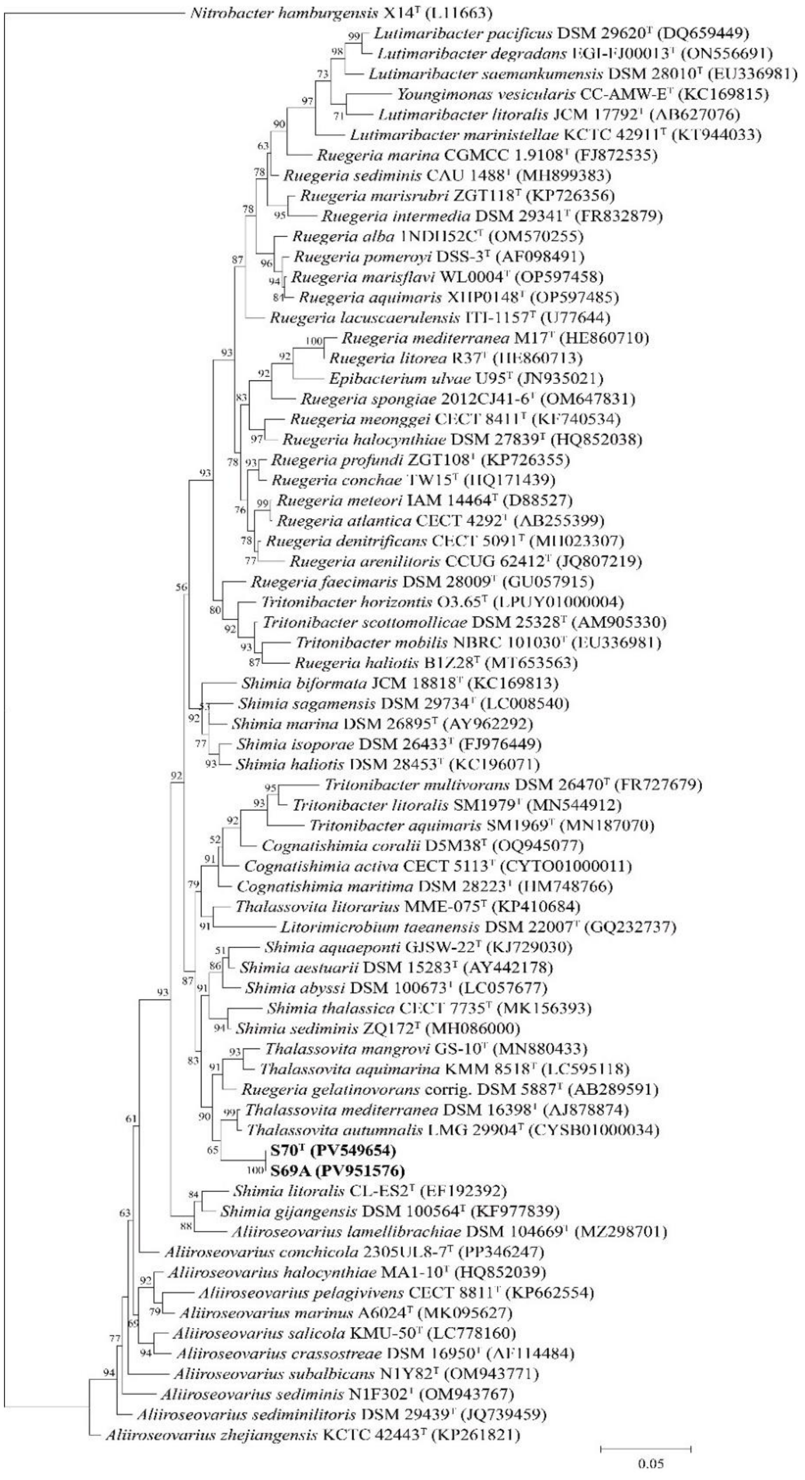

Supplementary Figure 3 Maximum-parsimony phylogenetic tree of 16S rRNA gene sequences, showing the position of strains S70^T and S69A among representatives of the *Roseobacteraceae*. Bootstrap values (above 50%) based on 1000 re-samplings are shown at branch nodes. *Nitrobacter_hamburgensis* X14^T (GenBank accession number, L11663) was used as an outgroup. Bar, 50 substitutions per nucleotide position.

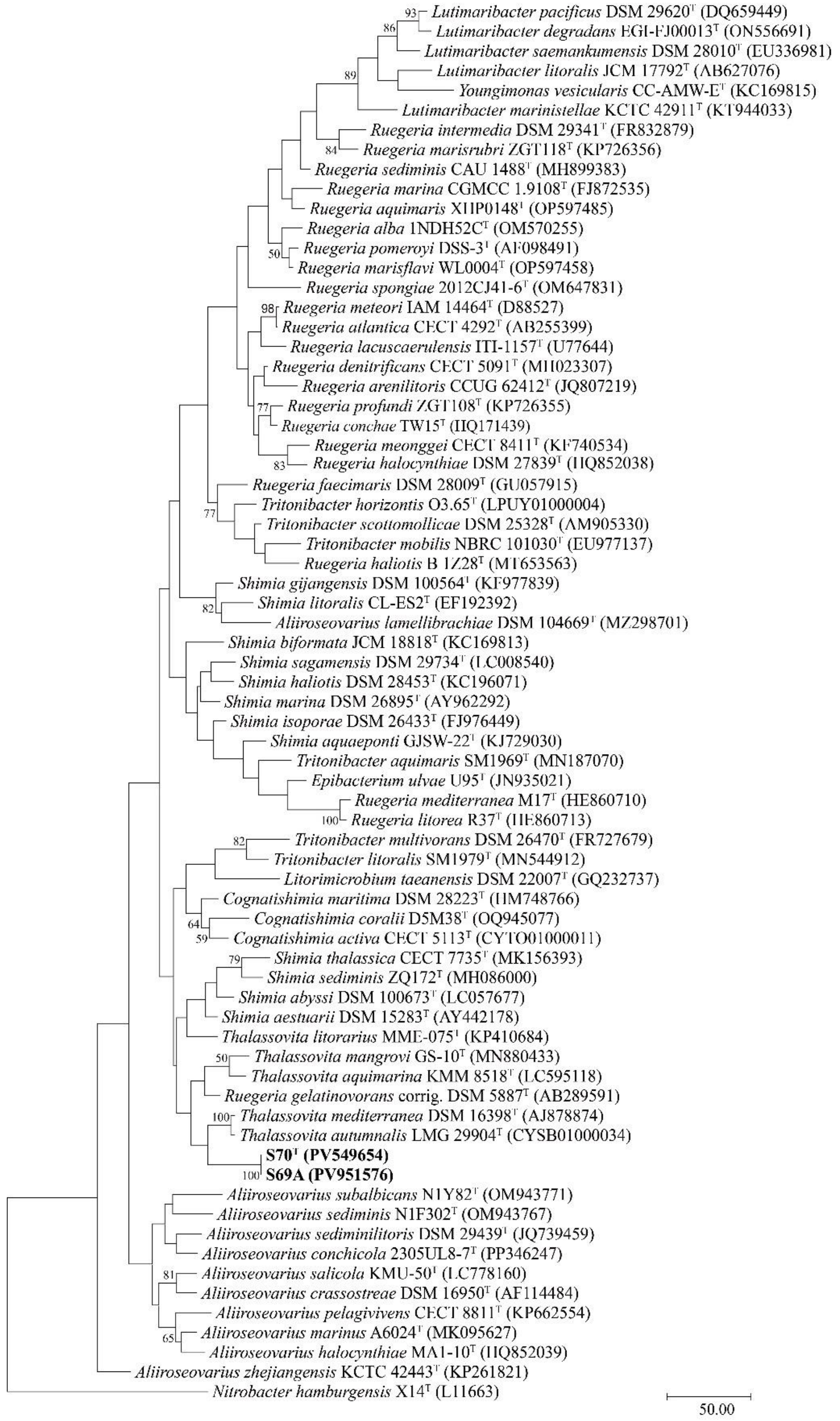

Supplementary Figure 4 Consensus trees based on the super-matrix (SM) trees of the gene sets rhodo268 and rp2, respectively.

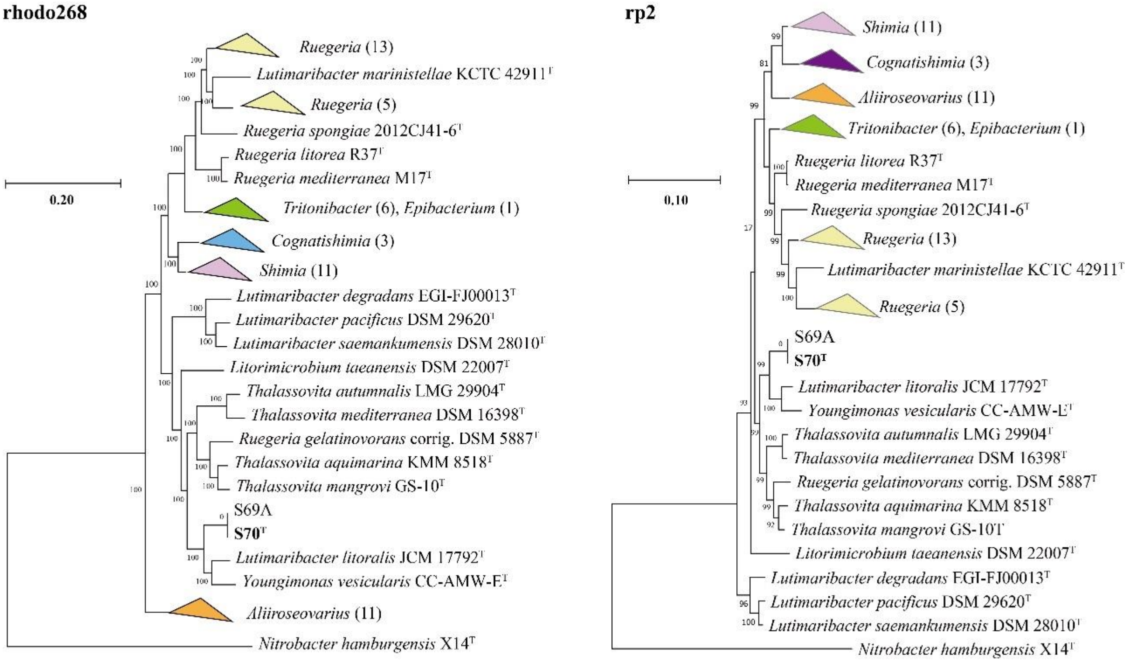

Supplementary Figure 5 The pyANIb heatmap with hierarchical clustering was constructed using genomic data retrieved from the NCBI database. *Nitrobacter hamburgensis* X14^{T} was selected as the outgroup for phylogenetic comparison. The red box highlights the cluster includes strain S70^{T} and its closest phylogenetic relatives among the type strains.

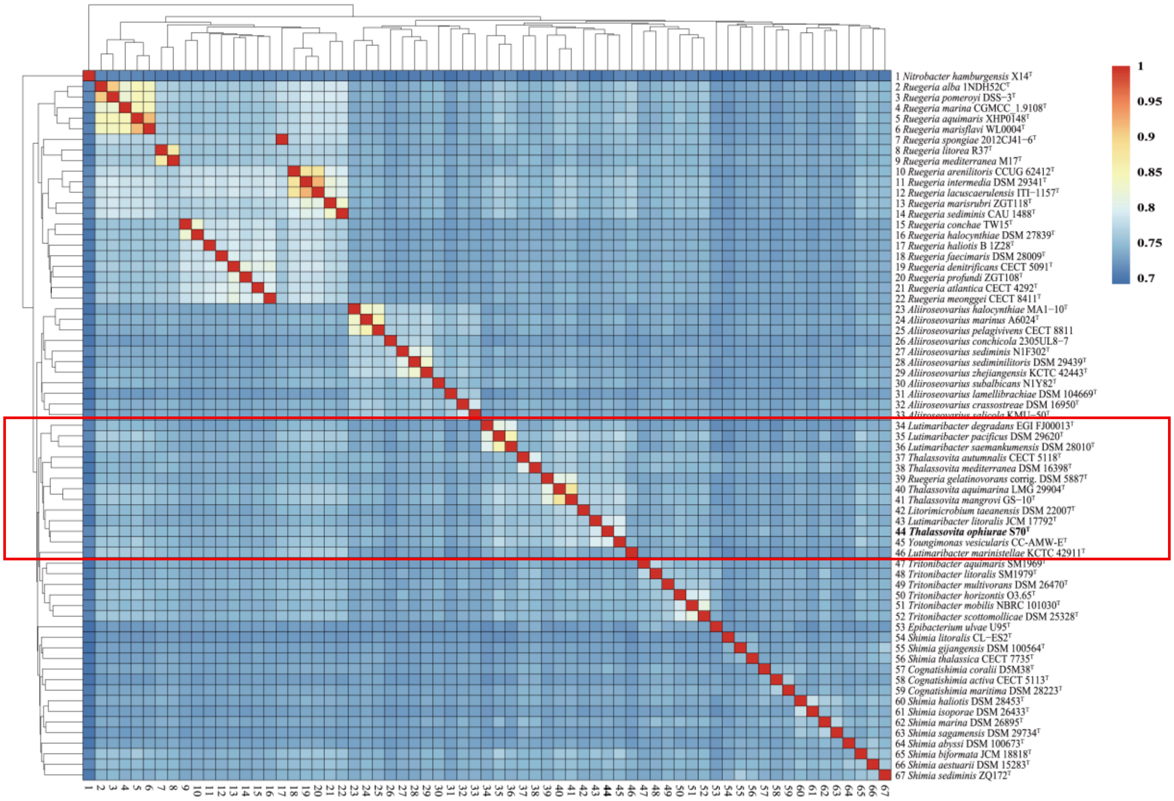

Supplementary Figure 6 The ezAAI-based heatmap with hierarchical clustering was generated using genomic data obtained from the NCBI database. *Nitrobacter hamburgensis* X14^{T} was selected as the phylogenetic outgroup. The red box highlights the cluster that includes strain S70^{T}, S69A and their closest phylogenetic relatives among the type strains.

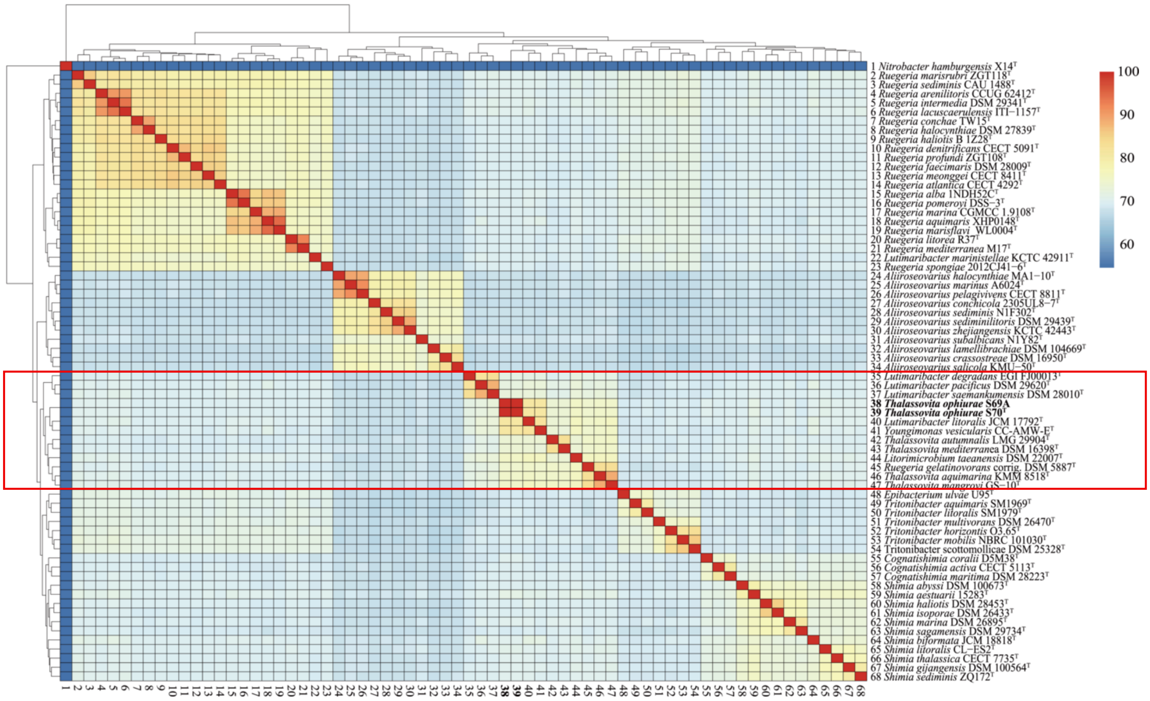

Supplementary Figure 7 Polar lipids of strain S70^T separated by two-dimensional TLC.

The polar lipids were detected with (a) molybdatophosphoric acid reagent, (b) molybdenum blue reagent, (c) ninhydrin reagent, and (d) anisaldehyde reagent. Chloroform/methanol/water (65:25:4 by vol.) was used in the first direction, followed by chloroform/acetic acid/methanol/water (80:15:12:4 by vol.) in the second direction.

Abbreviations: PC, phosphatidylcholine; PG, phosphatidylglycerol; DPG, diphosphatidylglycerol; PE, phosphatidylethanolamine; PME, phosphatidylmonomethylethanolamine; AL, unidentified aminolipid; L, unidentified polar lipid. F-first dimension of TLC; S- second dimension of TLC.

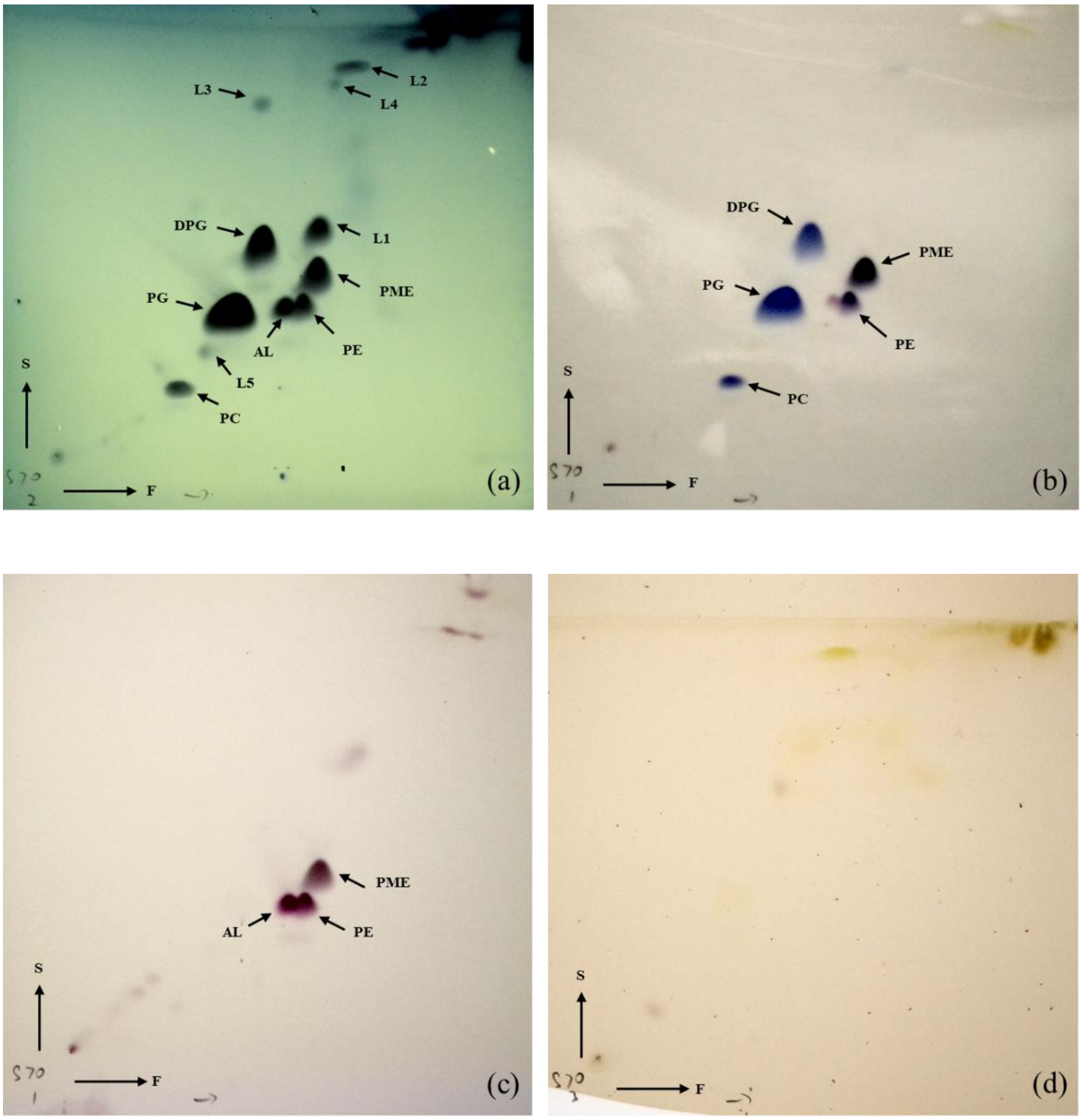

Supplementary Tables

Supplementary Table 1 Number of genes associated with the general COG functional categories for strain S70^{T}.

Function code	Number	Percentage	Function
C	234	7.34%	Energy production and conversion
D	53	1.66%	Cell cycle control, cell division, chromosome partitioning
E	335	10.51%	Amino acid transport and metabolism
F	102	3.20%	Nucleotide transport and metabolism
G	127	3.99%	Carbohydrate transport and metabolism
H	188	5.90%	Coenzyme transport and metabolism
I	238	7.47%	Lipid transport and metabolism
J	209	6.56%	Translation, ribosomal structure and biogenesis
K	214	6.72%	Transcription
L	119	3.74%	Replication, recombination and repair
M	190	5.96%	Cell wall/membrane/envelope biogenesis
N	46	1.44%	Cell motility
O	168	5.27%	Posttranslational modification, protein turnover, chaperones
P	144	4.52%	Inorganic ion transport and metabolism
Q	83	2.61%	Secondary metabolites biosynthesis, transport and catabolism
R	256	8.04%	General function prediction only
S	149	4.68%	Function unknown
T	138	4.33%	Signal transduction mechanisms
U	83	2.61%	Intracellular trafficking, secretion, and vesicular transport
V	57	1.79%	Defense mechanisms
W	21	0.66%	Extracellular structures
X	29	0.91%	Mobilome: prophages, transposons
Z	3	0.09%	Cytoskeleton

Supplementary Table 2 Genome assembly numbers of the strains used in this study. Quality analysis calculated on the Prokaryotic Genome Annotation Pipeline (PGAP) gene set using the *Roseobacteraceae* CheckM marker set.

Organism Name	Strain	GenBank	Coverage	Completeness (%)	Level
Youngimonas vesicularis	CC-AMW-E	GCA_004799325.1	200×	99.54%	Contig
Lutimaribacter litoralis	JCM 17792	GCA_900182725.1	131×	99.54%	Contig
Thalassovita autumnalis	LMG 29904	GCA_001458255.1	58×	99.39%	Scaffold
Thalassovita mediterranea	DSM 16398	GCA_900156595.1	438×	99.39%	Contig
Thalassovita aquimarina	KMM 8518	GCA_018219815.1	21×	99.24%	Contig
Thalassovita mangrovi	GS-10	GCA_009857745.1	311×	99.69%	Contig
Ruegeria gelatinovora	DSM 5887	GCA_900111045.1	267×	99.69%	Contig
Litorimicrobium taeanensis	DSM 22007	GCA_900110775.1	261×	99.39%	Scaffold
Lutimaribacter degradans	EGI FJ00013	GCA_023703375.1	1111×	99.08%	Contig
Lutimaribacter pacificus	DSM 29620	GCA_900142185.1	208×	99.39%	Contig
Lutimaribacter saemankumensis	DSM 28010	GCA_900100005.1	268×	98.93%	Contig
Lutimaribacter marinistellae	KCTC 42911	GCA_042648365.1	65×	99.69%	Scaffold
Aliiroseovarius crassostreae	DSM 16950	GCA_900116725.1	294×	99.71%	Scaffold
Aliiroseovarius marinus	A6024	GCA_004360145.1	399×	99.12%	Scaffold
Aliiroseovarius halocynthiae	MA1-10	GCA_007004645.1	412×	99.12%	Scaffold
Aliiroseovarius sediminilitoris	DSM 29439	GCA_900109955.1	310×	100%	Contig
Aliiroseovarius pelagivivens	CECT 8811	GCA_900302485.1	181×	98.83%	Contig
Aliiroseovarius subalbicans	N1Y82	GCA_022678625.1	150×	99.93%	Contig
Aliiroseovarius zhejiangensis	KCTC 42443	GCA_014656375.1	463×	100%	Contig
Aliiroseovarius salicola	KMU-50	GCA_027891095.1	151×	99.12%	Contig
Aliiroseovarius conchicola	2305UL8-7	GCA_041403445.1	150×	100%	Scaffold
Aliiroseovarius sediminis	N1F302	GCA_022678485.1	150×	99.71%	Contig
Aliiroseovarius lamellibrachiae	DSM 104669	GCA_018599245.1	100×	99.71%	Scaffold
Tritonibacter mobilis	NBRC 101030	GCA_030159715.1	65×	99.12%	Contig
Tritonibacter scottomollicae	DSM 25328	GCA_003003215.1	223×	99.41%	Scaffold
Tritonibacter horizontis	O3.65	GCA_001518015.1	71.5×	99.02%	Contig
Tritonibacter multivorans	DSM 26470	GCA_900112515.1	200×	99.41%	Scaffold
Tritonibacter litoralis	SM1979	GCA_009496005.1	739×	99.56%	Scaffold
Tritonibacter aquimaris	SM1969	GCA_009617595.1	679×	98.24%	Contig
Epibacterium ulvae	U95	GCA_002796795.1	130×	99.69%	Scaffold
Cognatishimia activa	CECT 5113	GCA_001458335.1	79×	97.95%	Scaffold
Cognatishimia maritima	DSM 28223	GCA_900129685.1	353×	99.71%	Scaffold
Cognatishimia coralii	D5M38	GCA_037478305.1	306×	98.83%	Contig
Shimia aestuarii	DSM 15283	GCA_900114635.1	252×	100%	Contig
Shimia litoralis	CL-ES2	GCA_005518135.1	286×	99.76%	Contig
Shimia isoporae	DSM 26433	GCA_004346865.1	193×	99.69%	Contig
Shimia biformata	JCM 18818	GCA_016888515.1	100×	100%	Scaffold
Shimia thalassica	CECT 7735	GCA_001458215.1	142×	99.69%	Contig
Shimia haliotis	DSM 28453	GCA_900114415.1	323×	100%	Contig

Shimia sagamensis	DSM 29734	GCA_900182745.1	172×	99.69%	Contig
Shimia sediminis	ZQ172	GCA_003990645.1	260×	99.39%	Contig
Shimia marina	DSM 26895	GCA_900112745.1	270×	98.03%	Scaffold
Shimia gijangensis	DSM 100564	GCA_900142085.1	184×	100%	Contig
Shimia abyssi	DSM 100673	GCA_003014475.1	205×	99.39%	Contig
Ruegeria pomeroyi	DSS-3	GCA_000011965.2	-	99.62%	Complete
Ruegeria conchae	TW15	GCA_000192475.2	44×	99.46%	Contig
Ruegeria halocynthiae	DSM 27839	GCA_900106805.1	172×	98.56%	Contig
Ruegeria atlantica	CECT 4292	GCA_001458195.1	107×	96.8%	Contig
Ruegeria marisrubri	ZGT118	GCA_001507595.1	12×	95.76%	Contig
Ruegeria sediminis	CAU 1488	GCA_005938215.1	519.6×	97.51%	Contig
Ruegeria denitrificans	CECT 5091	GCA_001458295.1	104×	97.65%	Contig
Ruegeria faecimaris	DSM 28009	GCA_900182615.1	167×	98.68%	Contig
Ruegeria aquimaris	XHP0148	GCA_025751555.1	120×	99.2%	Contig
Ruegeria marina	CGMCC 1.9108	GCA_900101475.1	188×	96.13%	Scaffold
Ruegeria haliotis	B 1Z28	GCA_013377785.1	2800×	98.31%	Scaffold
Ruegeria marisflavi	WL0004	GCA_025673445.1	120×	98.78%	Contig
Ruegeria alba	1NDH52C	GCA_022321465.1	200×	99.58%	Contig
Ruegeria spongiae	2012CJ41-6	GCA_023502395.1	1378.98×	98.57%	Contig
Ruegeria intermedia	DSM 29341	GCA_900129345.1	240×	98.09%	Scaffold
Ruegeria profundi	ZGT108	GCA_001507545.1	12×	97.59%	Contig
Ruegeria meonggei	CECT 8411	GCA_900172215.1	94×	97.65%	Contig
Ruegeria arenilitoris	CCUG 62412	GCA_042675705.1	76×	98.82%	Contig
Ruegeria lacuscaerulensis	ITI-1157	GCA_900141625.1	276×	-	Contig
Ruegeria mediterranea	M17	GCA_900302455.1	85×	99.39%	Contig
Ruegeria litorea	R37	GCA_900172225.1	68×	99.39%	Contig

Supplementary Table 3 Cellular fatty acid composition (%) of strain S70[T] and its phylogenetically closest strains.

Strain: 1, S70[T]; 2, *Y. vesicularis* JCM 18819[T]; 3, *L. litoralis* JCM 17792[T] 3, *T. autumnalis* LMG 29904[T]; 4, *T. mediterraneus* DSM 16398[T]; all data for four strains are from this study. Major fatty acids (>10%) are highlighted in bold type. TR, Trace (<1 %)

Fatty acids	1	2*	3	4	5
**Saturated:**					
$C_{9:0}$	0.2	–	0.1	–	–
$C_{10:0}$	–	TR	1.2	0.1	0.3
$C_{11:0}$	0.2	–	–	0.3	0.2
$C_{12:0}$	–	–	0.2	–	0.2
$C_{14:0}$	0.4	–	0.5	0.2	0.2
$C_{16:0}$	6.0	4.4	8.4	4.7	4.4
$C_{17:0}$	1.0	4.2	7.7	0.4	0.3
$C_{18:0}$	3.1	2.8	1.3	2.1	1.3
$C_{19:0}$	–	–	0.2	0.1	–
$C_{19:0}$ 11-methyl	–	–	–	–	0.2
Anteiso-$C_{11:0}$	0.1	–	–	–	–
Anteiso-$C_{15:0}$	–	–	–	0.1	0.2
Iso-$C_{10:0}$	–	–	0.5	–	–
Iso-$C_{11:0}$	–	–	0.3	–	0.2
Iso-$C_{13:0}$	–	–	0.2	–	–
Iso-$C_{14:0}$	–	–	–	0.2	–
Iso-$C_{17:0}$	–	–	0.1	–	–
**Unsaturated:**					
$C_{17:1}\omega 6c$	–	–	0.4	–	–
$C_{17:1}\omega 8c$	0.2	1.8	1.0	0.7	0.4
$C_{18:1}\omega 5c$	–	–	–	0.3	0.3
$C_{20:1}\omega 7c$	–	–	–	0.4	0.1
$C_{20:1}\omega 9c$	–	–	–	0.1	–
$C_{18:1}\omega 7c$ 11-methyl	5.8	TR	0.4	**11.5**	3.5
Iso-$C_{17:1}\ \omega 10c$	–	1.2	–	–	–
**Hydroxy:**					
$C_{10:0}$ 3-OH	0.5	—	0.7	2.3	2.8
$C_{11:0}$ 2-OH	–	—	0.4	TR	–
$C_{11:0}$ 3-OH	–	—	0.3	0.2	0.1
$C_{12:0}$ 3-OH	–	—	0.1	–	–
$C_{12:1}$ 3-OH	4.1	2.9	5.0	4.2	4.6
$C_{15:0}$ 2-OH	–	—	0.3	–	–
$C_{16:0}$ 2-OH	–	—	0.6	0.4	–
$C_{16:1}$ 2-OH	–	—	–	0.1	–
$C_{18:1}$ 2-OH	–	—	–	0.1	–
Iso-$C_{11:0}$ 3-OH	–	—	–	0.1	–
Iso-$C_{15:0}$ 3-OH	–	—	0.1	–	0.1
Sum feature 1*	–	—	0.7	–	–
Sum feature 2*	–	—	0.3	–	–
Sum feature 3*	3.3	5.3	3.0	0.6	0.9

	Sum feature 5*	–	–	–	TR	–
*	Sum feature 8*	**75.1**	**75.6**	**66.0**	**70.7**	**79.7**

Summed Features are fatty acids that cannot be resolved reliably from another fatty acid using the chromatographic conditions chosen. The MIDI system groups these fatty acids together as one feature with a single percentage of the total. Summed feature 1 contains iso-$C_{15:1}$ H/$C_{13:0}$ 3-OH; summed feature 2 contains iso-$C_{16:1}$ I/$C_{14:0}$ 3-OH and/or $C_{12:0}$ aldehyde; summed feature 3 contains $C_{16:1}$ *ω*6*c* and/or iso-$C_{16:1}$ *ω*7*c*; summed feature 5 contains $C_{18:2}$ *ω*6, 9*c* and/or anteiso-$C_{18:0}$; summed feature 8 contains $C_{18:1}$ *ω*6*c* and/or $C_{18:1}$ *ω*7*c*.

* Data from Hameed et al. [28]

Description of *Youngimonas ophiurae* sp. nov.

Youngimonas ophiurae (o.phi.u′ rae. N.L. gen. n. *ophiurae* of Ophiura, a class of invertebrates belonging to the Ophiuroidea, the source of isolation of the type strain).

Gram-stain-negative, rod shaped, approximately 0.4–0.6 μm in width and 1.1–5.2 μm in length; non-motile. After 3 days of incubation at 30 °C on MA, colonies are pale yellow, smooth and opaque. Growth occurs at 10–37°C (optimum at 24–30°C), pH 6.0–9.0 (opti-mum pH 7.0–8.0), and in the presence of 0.5-6% (w/v) NaCl (optimum at 2-3%). Strictly aerobic. Positive for catalase and oxidase activities, and for hydrolysis of Tween 20, 40, 60, L-tyrosine, and urea. Negative for hydrolysis of aesculin, DNA, casein, starch and gelatin. In the API 20NE system, nitrate is reduced to nitrogen, but acid production from assimilation test is negative. In API ZYM tests, positive reactions are observed for alkaline phos-phatase (weak), esterase (C4), esterase lipase (C8) (weak), leucine arylamidase, and naphthol-AS-BI-phosphohydrolase; negative for lipase (C14), valine arylamidase, cystine ar-ylamidase, trypsin, α-chymotrypsin, acid phosphatase, α-galactosidase, β-galactosidase, β-glucuronidase, α-glucosidase, β-glucosidase, N-acetyl-β-glucosaminidase, α-mannosidase and α-fucosidase. In the GENIII MicroPlate test, utilizes of dextrin, D-maltose, D-trehalose, gentiobiose, sucrose (weakly), D-turanose, stachyose, D-raffinose, α-D-glucose (weakly), myo-inositol, D-Fructose-6-PO_4, glycyl-L-proline, L-aspartic acid, L-glutamic acid (weakly), pectin, D-galacturonic acid (weakly), L-galactonic acid (weakly), glucuronamide, *p*-hydroxy-phenylacetic, Tween 40, acetoacetic acid, acetic acid, and for-mic acid. The major fatty acid is summed feature 8 (75.1%). The predominant ubiquinone is Q-10. The polar lipids profile comprises PG, DPG, PE, PME, PC, AL, and five unidentified polar lipids. The DNA G+C content of the type strain S70^T is 61.5%. The ANIb, OrthoANIu, and dDDH values between strain S70^T, S69A and each reference strain were below 95–96%, 95% and 70%, respectively. The type strain, S70^T (=KCTC 8975^T =MCCC 1K09707^T), was isolated from a brittle star collected in the South China Sea, Shenzhen, China.
